# Revolutionizing spine surgery with emerging AI–FEA integration

**DOI:** 10.1007/s11701-025-02772-w

**Published:** 2025-09-18

**Authors:** Christopher Franceschini, Mohsen Ahmadi, Xuanzong Zhang, Kelly Wu, Maohua Lin, Ridge Weston, Angela Rodio, Yufei Tang, Erik Engeberg, Gui Pires, Talha S. Cheema, Frank D. Vrionis

**Affiliations:** 1 College of Medicine, Florida Atlantic University, Boca Raton, FL USA; 2 Department of Electrical and Computer Science, Florida Atlantic University, Boca Raton, FL USA; 3 Department of Biomedical Engineering, Florida Atlantic University, 777 Glades Rd, Bldg. 36, Room 273, Boca Raton, FL 33431 USA; 4 Department of Mechanical and Ocean Engineering, Florida Atlantic University, Boca Raton, FL USA; 5 Department of Neurosurgery, Marcus Neuroscience Institute, Boca Raton Regional Hospital, 800 Meadows Road, Boca Raton, FL USA; 6 Department of Surgery, Schmidt College of Medicine, Florida Atlantic University, Boca Raton, USA

**Keywords:** Artificial intelligence, Finite element analysis, Spine surgery, Digital twin, Surgical planning, Intraoperative navigation, Postoperative prediction

## Abstract

This study explores the integration of artificial intelligence (AI) and finite element analysis (FEA) in spine surgery, highlighting their complementary roles across preoperative planning, intraoperative execution, and postoperative outcome prediction. The synergy between AI and FEA is reshaping modern spine care by improving biomechanical modeling, enhancing surgical precision, and enabling personalized treatment strategies. In the preoperative phase, AI-augmented FEA supports the design of patient-specific surgical plans, optimizing implant placement and simulating mechanical responses under various loading conditions. Intraoperatively, AI enables real-time image-guided navigation, robotic assistance, and automated anatomical recognition, reducing the risk of surgical error. Postoperatively, predictive models built on FEA simulations and patient data assist in tracking recovery, forecasting complications, and informing rehabilitation protocols. Together, these technologies contribute to a data-driven paradigm shift toward precision spine surgery. As intelligent feedback systems, digital twins, and autonomous surgical platforms continue to evolve, AI–FEA integration is poised to play a transformative role in delivering safer, more efficient, and individualized spine care.

## Introduction

Spinal disorders represent a significant global health challenge, affecting approximately 4.9% of the population, with a notably higher prevalence among women [12]. These conditions are among the leading causes of disability worldwide and contribute substantially to healthcare utilization. Between 25 and 30% of all medical referrals and approximately 15% of global medical certifications are attributed to spinal pathologies, with associated annual costs exceeding $100 billion in the United States alone [71]. The burden is expected to increase with aging populations and rising life expectancy, particularly impacting males and older adults in low-income regions. The spectrum of spinal disorders, including degenerative disc disease, scoliosis, spinal cord injuries, and complex deformities, presents diverse diagnostic and therapeutic challenges [40]. Additionally, socioeconomic disparities, limited surgical expertise in resource-constrained regions, and unequal access to rehabilitation services further complicate treatment outcomes [16].

Over the past decades, spine surgery has undergone a significant transformation, shifting from invasive, generalized procedures to more refined, patient-specific interventions. Innovations, such as intraoperative imaging, robotic assistance, and real-time surgical navigation, have markedly improved procedural safety, minimized soft-tissue damage, and accelerated recovery [119]. Minimally invasive techniques, such as minimally invasive transforaminal lumbar interbody fusion (MIS-TLIF), have demonstrated notable advantages over traditional open TLIF, including reduced intraoperative blood loss and shorter hospital stays [131]. Concurrently, advances in orthopedic materials science have further enhanced surgical outcomes. Metals like titanium and stainless steel continue to be widely used due to their strength and biocompatibility; however, concerns related to inflammation and ion leaching have prompted the adoption of polymers such as polyether ether ketone (PEEK) and polylactic acid (PLA) for fixation devices and interbody cages [27, 115, 144]. These materials are radiolucent, reducing imaging artifacts during follow-up, and offer mechanical properties more akin to natural bone. Moreover, ceramics and bioresorbable materials present additional options with enhanced osteointegration and customizable biomechanical behavior [142]. While these innovations have significantly advanced spine care, the integration of artificial intelligence (AI and physics-based biomechanical simulations)—particularly finite element analysis (FEA)—represents the next frontier. These emerging tools promise to enable more personalized, predictive, and efficient surgical planning and therapeutic outcomes.

### The promise of AI in spinal surgery

Artificial Intelligence (AI), particularly through machine learning (ML) and deep learning (DL), is rapidly emerging as a transformative force in modern healthcare. In spinal surgery, where millimeter-scale precision can critically impact patient outcomes, AI offers significant advantages in navigating the complex anatomy of the spine. It has demonstrated robust performance in medical imaging interpretation, disease classification, outcome prediction, and intraoperative guidance [1, 12]. Initial barriers to widespread AI adoption in spinal care, such as data scarcity, variability in imaging protocols, and the anatomical intricacy of the spine, have been gradually overcome. Notably, approximately 86% of spine-related AI publications have been published since 2017, indicating exponential growth in this field [34]. Table 1 provides a structured overview of the primary applications of artificial intelligence (AI) across different stages of spine surgery. It categorizes AI implementation into four key surgical phases: diagnosis, preoperative planning, intraoperative execution, and postoperative monitoring highlighting how AI technologies, particularly convolutional neural networks (CNNs) and augmented reality (AR), are transforming clinical workflows.

**Table 1:** Applications of artificial intelligence in spine surgery

Surgical stage	Application	Description
Diagnosis	Image interpretation	CNNs detect stenosis, fractures, tumors, and degeneration in CT/MRI scans
Preoperative planning	Surgical path simulation	AI simulates optimal screw trajectories and surgical strategies
Intraoperative execution	Robotic assistance and AR navigation	Enhances precision via real-time guidance and augmented visual overlays
Postoperative monitoring	Outcome prediction and rehab guidance	AI models predict recovery trends and personalize rehabilitation protocols

Neural networks, especially convolutional neural networks (CNNs), have proven capable of detecting spinal stenosis, fractures, tumors, and disc degeneration with high accuracy in MRI and CT scans [6, 40]. AI applications in spine surgery can be broadly categorized into four stages: (1) diagnosis, including detection of spinal pathologies and grading of disease severity; (2) preoperative planning, where AI simulates optimal screw trajectories and recommends surgical strategies [35, 78], (3) intraoperative execution, enhancing precision through robotic systems and augmented reality tools [7], and (4) postoperative monitoring, where AI models help track recovery and tailor rehabilitation protocols.

### Role of finite element analysis (FEA)

Finite Element Analysis (FEA) has been foundational in spine biomechanics. By decomposing spinal anatomy into a mesh of discrete elements, FEA enables the calculation of stresses, strains, and displacements under various physiological and pathological loading conditions [34]. Applications range from implant testing to surgical simulation and material evaluation. However, traditional FEA is time-consuming, computationally expensive, and relies on manual steps for segmentation, meshing, and property assignment [34]. This makes it impractical for fast-paced clinical environments. The integration of AI with FEA solves many of these issues. AI can automate geometry segmentation, optimize mesh generation, and predict material properties, enabling faster, personalized modeling [4, 39]. A major advancement is the use of Physics-Informed Neural Networks (PINNs), which embed biomechanical equations into the network structure, enabling simulations that are both data- and physics-driven. This is particularly useful for estimating spinal loads, disc degeneration behavior, and surgical outcomes [89]. AI is revolutionizing not just spinal surgery, but multiple domains of medicine.

**Fig. 1:**
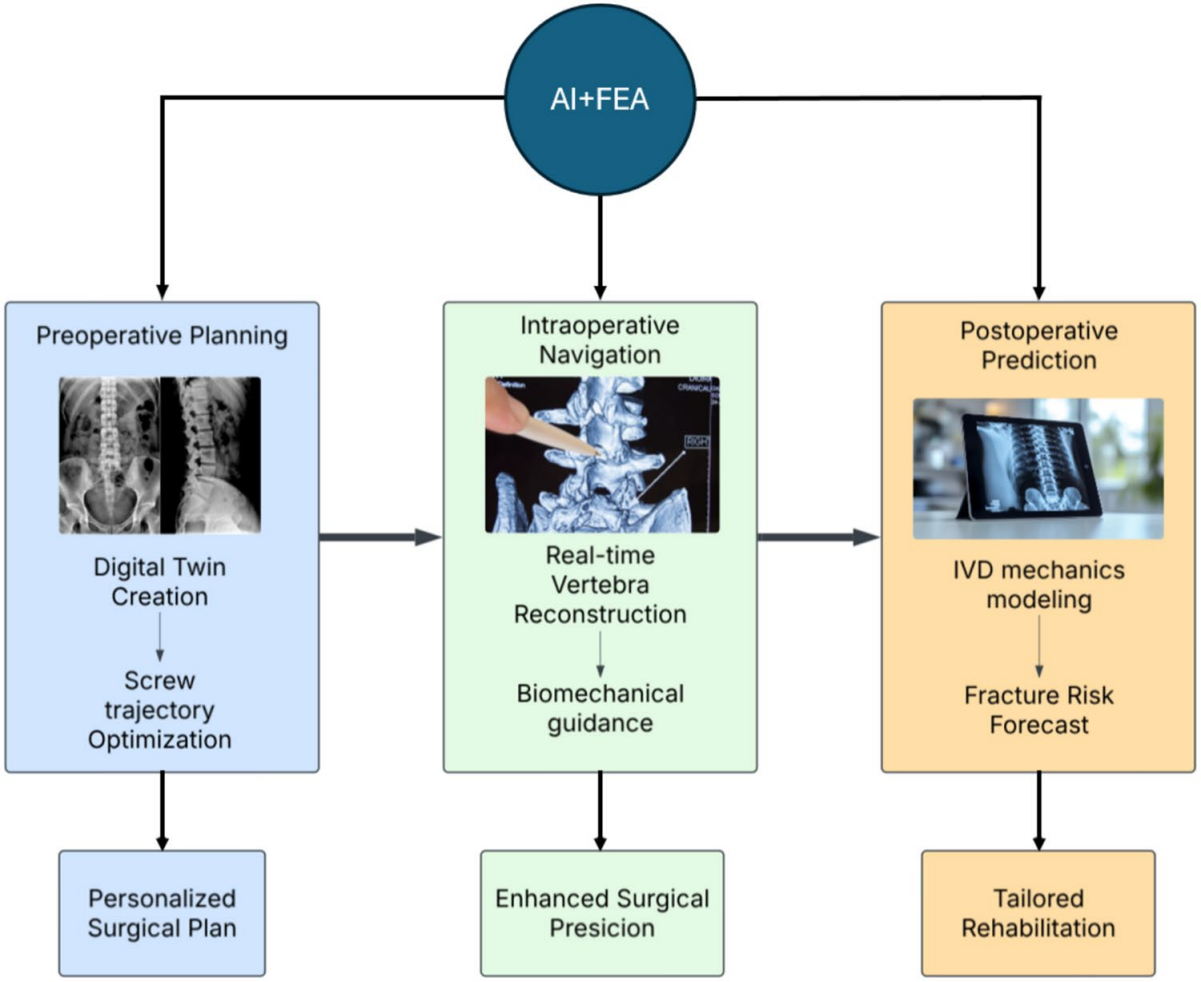
Workflow of AI–FEA integration in spine surgery

Traditional implants are mass-manufactured based on generalized anatomical data. While functional, this approach often fails to accommodate patient-specific geometries, leading to alignment issues and longer recovery times [90]. Additive manufacturing (3D printing) allows for rapid prototyping and the production of implants tailored to individual patients [101]. Materials like titanium, PEEK, and hybrid composites are being used to fabricate vertebral cages, pedicle screws, and artificial discs with high mechanical fidelity [45, 156]. Bioprinting, using cell-laden bioinks, is also being explored for regenerating spinal discs and intervertebral tissues. AI contributes to 3D printing by optimizing design parameters, such as porosity, stiffness gradients, and trabecular architecture [10]. When combined with patient-specific imaging and FEA, these technologies enable fully customized surgical solutions. Recent studies categorize AI spine applications into six clinical areas [34]. Figure 1 illustrates the AI–FEA integration workflow across the spine surgery continuum (preoperative, intraoperative, and postoperative phases), highlighting key applications like digital twin creation, real-time navigation, and fracture risk forecasting. Created in Lucid (lucid.co). AI enhances FEA by automating labor-intensive tasks, such as generating patient-specific models from imaging data, and optimizing simulations to predict biomechanical outcomes like spinal stability or implant performance. For example, AI can streamline the design of surgical plans tailored to an individual’s anatomy, improving precision and reducing risks compared to traditional methods. Figure 1 illustrates this workflow, highlighting how AI–FEA collaborate to enhance outcomes at each stage [59, 99].

AI applications in spinal imaging and surgical planning have expanded into six key clinical domains. First, image improvement techniques enhance CT and MRI resolution while simultaneously reducing the required radiation dose, making imaging safer and more efficient. Second, AI supports diagnosis by accurately detecting spinal curvature abnormalities, vertebral fractures, and implanted hardware. Third, in disease grading, AI models assist clinicians by classifying conditions, such as spinal stenosis, disc degeneration, and ossification of the posterior longitudinal ligament (OPLL) with high precision. Fourth, surgical simulation capabilities allow AI to recommend optimal screw trajectories and even generate synthetic CT images for planning and training purposes. Fifth, AI enables opportunistic diagnosis, where routine imaging is analyzed to uncover hidden risk factors such as low bone density or muscle degeneration that might otherwise go unnoticed. Finally, in decision support, AI systems predict potential postoperative complications and help design individualized treatment plans, contributing to more informed, personalized, and data-driven spine care.

Spinal care is moving toward intelligent systems driven by continuous data streams. Digital twins—real-time computational models of patient anatomy—will allow dynamic simulations of surgical outcomes and rehabilitation [16]. Large language models (LLMs) are being integrated into surgical support systems to generate clinical reports and triage responses [34]. Physics-informed models like PINNs will further bridge biomechanical reality with learning efficiency, reducing reliance on large datasets. Meanwhile, explainable AI (XAI) will make model outputs transparent and trustworthy, enhancing clinical adoption [1, 131].

### Challenges and contribution

Despite promising progress, challenges remain. There is no universally accepted dataset for training AI in spine imaging or biomechanics, leading to reproducibility issues. Many tools remain experimental and are not FDA-approved for clinical use [12]. Ethical concerns include data bias, privacy risks, and transparency of AI recommendations. Furthermore, advanced 3D-printed materials require long-term validation under in vivo conditions [115]. Equitable access in low-resource settings is also a significant challenge. This review offers a comprehensive, multidisciplinary synthesis of the role of A-FEA in modern spinal surgery. The major contributions include:

- Explaining how AI enhances diagnostics, planning, and monitoring
- Detailing FEA’s biomechanics and its limitations
- Introducing the AI–FEA hybrid for precision modeling
- Highlighting material and manufacturing innovations
- Presenting ethical and regulatory issues for real-world deployment.

We aim to provide a roadmap for the future of intelligent spinal care—merging clinical precision with computational intelligence. This paper serves researchers, clinicians, and technologists to reduce complications, lower costs, and enhance outcomes in spine surgery.

## AI–FEA synergy in spine surgery

The convergence of artificial intelligence (AI) and finite element analysis (FEA) is revolutionizing spine surgery by offering a more precise, data-driven approach to surgical planning and biomechanical evaluation. FEA has long served as a foundational method for analyzing how spinal components respond to various mechanical forces. However, the traditional workflow for developing FEA models, such as manually segmenting CT/MRI data, creating meshes, and assigning material properties, is time-consuming and often requires expert input [4]. AI simplifies and enhances this process through deep learning models that can automate anatomical segmentation, predict patient-specific biomechanical properties, and generate high-quality computational meshes [78]. This synergy leads to faster, more personalized simulations that support clinicians in planning complex interventions such as spinal deformity correction or disc prosthesis placement. Artificial intelligence and machine learning are reshaping spine research and practice. Galbusera et al. [21] reviewed their applications, ranging from image segmentation and computer-aided diagnosis to biomechanics and outcome prediction, laying a foundation for their integration with finite element analysis to optimize surgical outcomes. The collaboration between AI–FEA revolutionizes spine surgery by enabling personalized, data-driven interventions across all surgical phases preoperative planning, intraoperative execution, and postoperative recovery [35].

**Fig. 2:**
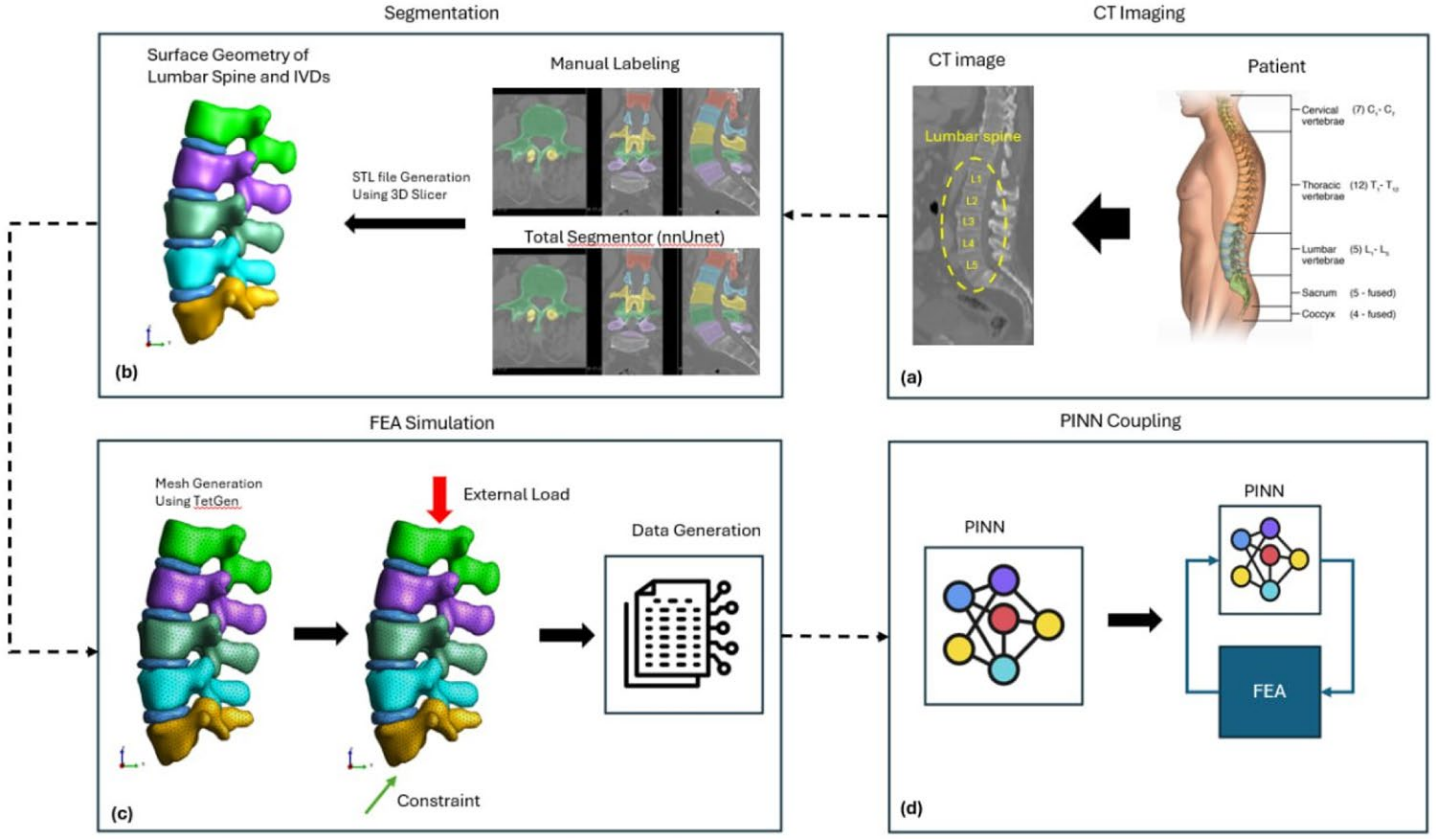
Workflow demonstrating the synergy between artificial intelligence (AI) and finite element analysis (FEA) in spine surgery [4]

Figure 2 illustrates the synergistic connection between AI–FEA in spine surgery.

(a) CT imaging of the patient’s lumbar spine serves as the anatomical foundation. (b) Segmentation of spinal components (vertebrae and intervertebral discs) is carried out through manual labeling or automated tools like TotalSegmentator (nnUNet), followed by 3D reconstruction and STL file generation. (c) FEA simulation includes mesh generation (using TetGen), application of external loads and boundary constraints, and generation of mechanical response data. (d) Coupling with Physics-Informed Neural Networks (PINNs) enables the integration of FEA-generated data with AI models, enhancing simulation accuracy, enabling real-time predictions, and creating a feedback loop ultimately supporting personalized spine surgery planning and analysis.

Table 2 presents a comparative overview of Artificial Intelligence in Surgery (AI in S) and Artificial Intelligence Surgery (AIS). The key distinction lies in the level of surgeon involvement and the role of AI in the surgical process. In AI in S, the surgeon maintains full control, while AI serves as an assistive tool, supporting various aspects of surgery, such as modeling, simulation, decision-making, safety monitoring, and partial automation. This represents the current state of technology, characterized by ongoing development and clinical integration. In contrast, AIS refers to a hypothetical future concept in which the surgeon’s role is minimal or potentially absent. Here, AI is envisioned to autonomously perform surgical tasks, make independent intraoperative decisions, and manage safety systems without human intervention. While AI in S enhances the surgeon’s capabilities, AIS aims to replace them in selected scenarios, raising complex questions about autonomy, safety, and ethical responsibility.

**Table 2:** Comparison between AI in surgery (AI in S) and artificial intelligence surgery (AIS)

Feature	Artificial intelligence in surgery (AI in S)	Artificial intelligence surgery (AIS)
Surgeon involvement	High (surgeon in control)	Low (potential future scenario with minimal or no surgeon involvement)
Current state	Existing technology with ongoing development	Hypothetical future concept
Role of AI	Assist surgeons in various aspects of surgery	Potentially perform surgery autonomously
Modeling for prediction	AI-driven modeling aids in understanding surgical processes and predicting outcomes	Models may be used to guide autonomous decision-making based on predictive insights
Simulation	Used in digital twins and AR to support planning and risk stratification	Could simulate procedures independently before autonomous execution
Decision-making assistance	Supports intraoperative decisions and workflow optimization	May replace surgeon decision-making in future autonomous systems
Ensuring safety	Monitors intraoperative safety (e.g., OR Black Box), and postoperative surveillance	Could integrate full digital safety and response systems
Automation	Automates specific tasks (e.g., suturing via robots like STAR)	Aims to automate complete procedures without human intervention

Beyond preoperative modeling, the integration of AI with FEA extends its benefits to intraoperative guidance and postoperative outcome prediction. By leveraging AI’s capacity to learn from large datasets and FEA’s ability to simulate realistic mechanical behavior, these hybrid models can predict surgical outcomes, such as implant stability, stress distribution, and healing potential. Advanced AI techniques like Physics-Informed Neural Networks (PINNs) are especially promising, as they can infer complex material behavior and simulate spinal biomechanics with minimal training data [4]. This makes it possible to create real-time simulations that guide surgical decisions, offering a form of "virtual prototyping" for personalized procedures [21].

As the integration of AI–FEA continues to mature, it is transforming spine surgery into a more predictive and patient-specific discipline. Surgeons can now evaluate multiple surgical strategies on a virtual spine model that mirrors the patient’s anatomy and pathology, ultimately reducing intraoperative risks and improving postoperative outcomes. For instance, models developed with AI-enhanced FEA have already been used to predict pseudoarthrosis risks, optimize screw trajectories, and assess fusion quality. These innovations are not only improving clinical decision-making but are also paving the way for digital twin technology in spine care, where simulations continually adapt and refine treatment in response to real-world patient data.

## Digital twin modeling and biomechanical simulations

Digital twin modeling in spine surgery creates virtual replicas of a patient’s spine using imaging techniques like MRI or CT scans. These detailed 3D models mirror the patient’s unique spinal anatomy, offering a personalized approach to surgical planning. Artificial intelligence (AI), particularly deep learning algorithms, processes the imaging data to build these models quickly and accurately [40]. Once constructed, finite element analysis (FEA) simulates how the spine behaves under different forces, providing insights for preoperative planning and risk assessment. The increasing prevalence of spinal disorders, such as low back pain (LBP) and scoliosis, highlights the urgent need for improved biomechanical assessment and predictive tools [16]. Modern lifestyles involving prolonged sitting, poor posture, and excessive use of digital devices contribute significantly to spinal degeneration. In clinical settings, traditional imaging techniques like CT and MRI help diagnose structural abnormalities but are limited to static evaluations, failing to capture the dynamic behavior of the spine during motion.

This gap presents a critical challenge for both diagnosis and intervention planning, particularly when considering the biomechanical consequences of conditions like lumbar disc herniation, facet joint degeneration, and scoliosis progression [16]. Recent advancements in digital twin technology and artificial intelligence (AI) offer a promising avenue for real-time monitoring and simulation of spinal biomechanics. A digital twin represents a virtual, adaptive model of the human spine that integrates physics-based simulations (e.g., finite element analysis) and data-driven AI models. This enables continuous tracking of spinal behavior under various physiological conditions. Smart wearable devices, such as inertial sensors and optical fiber systems, allow real-time capture of spine movement, which can be fed into AI algorithms to estimate key biomechanical variables such as facet contact forces and intradiscal pressures [35]. Such integration supports predictive modeling and personalized treatment planning for spine-related conditions, including scoliosis and chronic LBP [40].

**Fig. 3:**
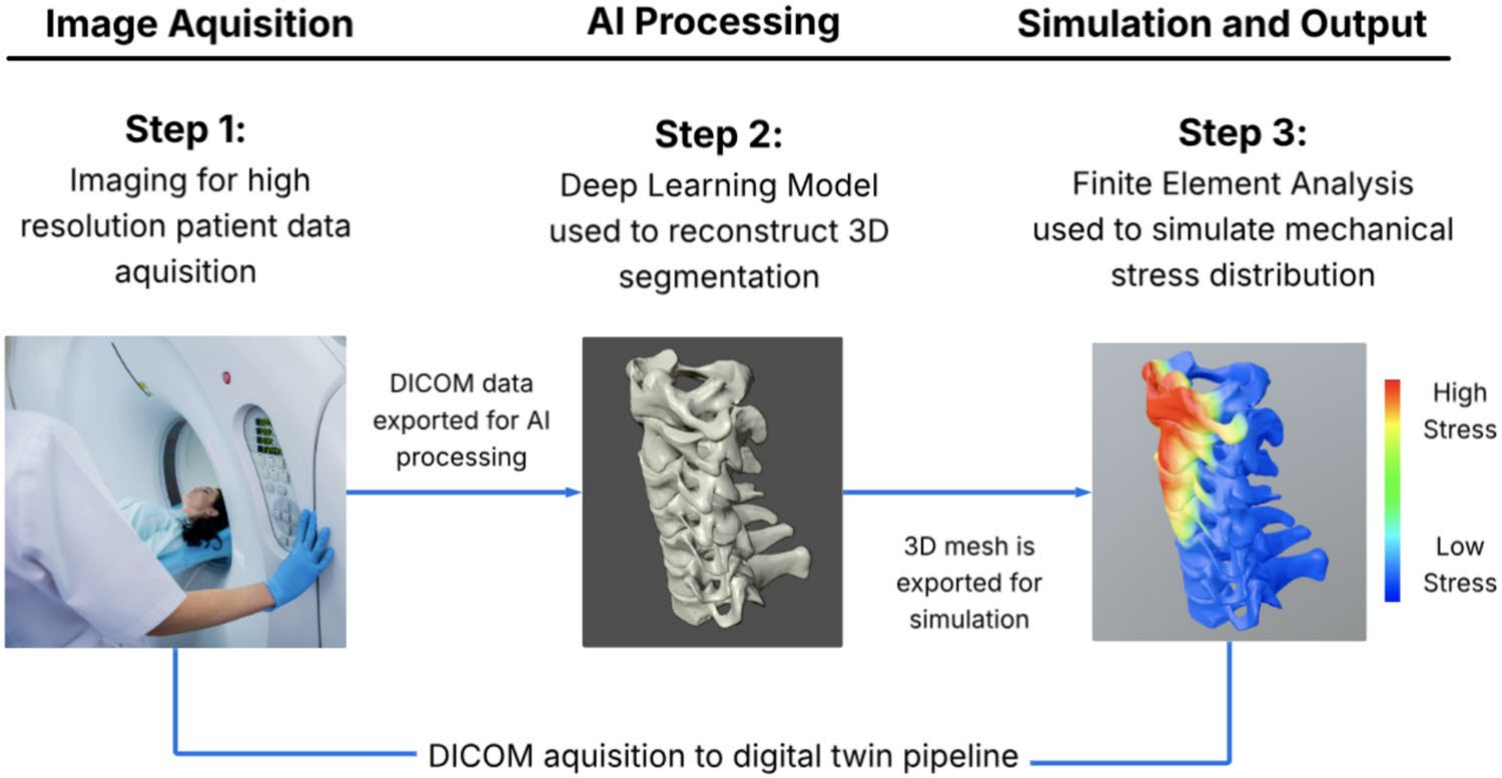
Digital twin creation process

Figure 3 illustrates the digital twin creation pipeline in spine surgery: (1) image acquisition using CT or MRI to obtain high-resolution patient-specific data, (2) AI processing with deep learning for 3D spine reconstruction, and (3) simulation and output via Finite Element Analysis (FEA) to simulate biomechanical behavior for surgical planning. Created with Blender and Lucid (lucid.co). Several studies highlight the power of this technology. Liang et al. [78] used a modified U-Net, a deep learning model, to automate lumbar spine modeling. Their method achieved a 97.8% Dice similarity coefficient—indicating near-perfect replication of the spine’s structure—and generated personalized FEA models in about 10 min. These models help surgeons predict outcomes, such as how loads distribute after placing implants, enhancing surgical precision. Further advancing digital twin technology, Ahmadi et al. [4] integrated finite element analysis with physics-informed neural networks (PINNs) to model the lumbar spine. Using CT and MRI scans, their method automates segmentation and meshing of vertebrae and intervertebral discs while predicting material properties—such as Young’s modulus and Poisson’s ratio—with 94.30% accuracy. This approach reduces manual effort and enhances the biomechanical fidelity of patient-specific models, offering a robust tool for preoperative planning and risk assessment.

Ahmadian et al. [5] developed ReconGAN, a tool based on a 3D Deep Convolutional Generative Adversarial Network (DCGAN). Trained on just 5 cuboid specimens from a single cadaveric vertebra, ReconGAN synthesized trabecular bone structure with less than 2% mean error, confirmed by two-point correlation functions. Their FEA simulations showed a 28% load reduction in lytic lesions and a 13% reduction in blastic lesions, offering valuable data for understanding vertebral strength. Tajdari et al. [137] created a patient-specific finite element model for adolescent idiopathic scoliosis, integrating AI to predict spinal curvature progression. This approach supports early intervention by enabling surgeons to anticipate curve severity and tailor treatment plans. Focusing on intervertebral discs (IVDs), Muñoz-Moya et al. [40] created 169 lumbar IVD models from MRI scans. Validated against ex vivo testing, these models achieved over 92% shape similarity, paving the way for accurate simulations of disc mechanics during surgery or recovery. Jecklin et al. [63] took a different approach, using domain adaptation to reconstruct 3D lumbar spine models from real fluoroscopic images. Their technique scored an 84% *F*1 score, demonstrating reliable identification of spinal features. This method supports real-time model creation, potentially aiding FEA simulations during operations.

Recent studies have advanced the integration of AI–FEA in spine surgery. Lomax et al. [59] highlighted the growing role of digital twin technologies in facilitating personalized preoperative and postoperative strategies. Singh et al. [16] and Luan and Morgan [34] contributed to improving patient-specific modeling, while Zheng et al. [136] focused on enhancing physiological accuracy each maintaining high fidelity to original anatomical and clinical data. Despite these advancements, several challenges remain in translating these models into routine clinical practice. For instance, Garavelli et al. [35] validated a patient-specific lumbar spine FEA model using digital image correlation, demonstrating strong experimental agreement. However, the study emphasized the need for thorough model verification and uncertainty quantification to ensure clinical reliability.

Similarly, Molinari et al. [39] investigated the biomechanical implications of pedicle screw angulation, identifying the caudomedial trajectory as the safest. Nevertheless, their findings were limited by assumptions in fracture modeling and a lack of quantitative validation, underscoring the importance of rigorous testing before digital twin technologies can be widely adopted in clinical spine surgery. While significant progress has been made, challenges remain in making digital twin systems clinically viable. Many existing spine modeling efforts are either static or overly reliant on specialized equipment like virtual reality motion sensors, making them less accessible in routine practice. Additionally, current models often lack sufficient clinical validation or generalizability across diverse patient populations [16]. To advance the field, future research should focus on creating robust, validated digital twin frameworks that can operate in real-time using standard clinical data and wearable sensors. These systems could revolutionize spine care by enabling early diagnosis, predicting disease progression, and informing targeted, patient-specific therapies [59].

## AI-enhanced surgical planning

Artificial intelligence (AI) integrated with finite element analysis (FEA) transforms preoperative planning into spine surgery by delivering precise biomechanical insights that enhance stability and minimize risks. For example, Ma et al. [35] developed a machine learning-based FEA system that optimizes pedicle screw trajectories in osteoporotic spines. This approach resulted in a 2.0–4.6× increase in pull-out force compared to traditional methods, demonstrating the AI’s ability to analyze complex spinal geometries and loading conditions. Clinically, this translates to reduced risks of fixation failure, fewer revision surgeries, and improved long-term patient outcomes, such as enhanced spinal stability and reduced postoperative pain. Zhou and Willing [137] utilized ANNs and multiobjective optimization to refine a biconcave mobile-bearing artificial disc design. Balancing ROM, FJF, and PCP through FEA, their approach aims to enhance TDA outcomes, demonstrating AI’s role in preoperative implant optimization.

Similarly, Phellan Aro et al. [92] used random forests to predict postoperative spinal alignment with an RMSE of 3.75 ± 0.56 mm, enabling surgeons to anticipate and address potential alignment issues before surgery. This model employed random forests, an ensemble of decision trees with optimized hyperparameters (e.g., tree depth, number of trees), to capture nonlinear relationships between preoperative variables and postoperative outcomes. The high predictive accuracy allows for tailored interventions that improve patient mobility and reduce complications like adjacent segment degeneration. Other studies further demonstrate the capabilities of AI–FEA in preoperative planning. Jimenez et al. [16] simulated spinal cord compression in traumatic spinal cord injury (SCI), achieving an area under the curve (AUC) of 0.79–0.82. Tajdari et al. [136] predicted adolescent idiopathic scoliosis (AIS) curvature with errors ranging from 0.60 to 10.43%. Peng et al. [78] developed a neural network-based tool to optimize the proximal junction angle in scoliosis surgery, predicting postoperative proximal junctional kyphosis risk with 83.3% accuracy. This personalized approach aids surgeons in tailoring surgical plans to minimize complications.

**Fig. 4:**
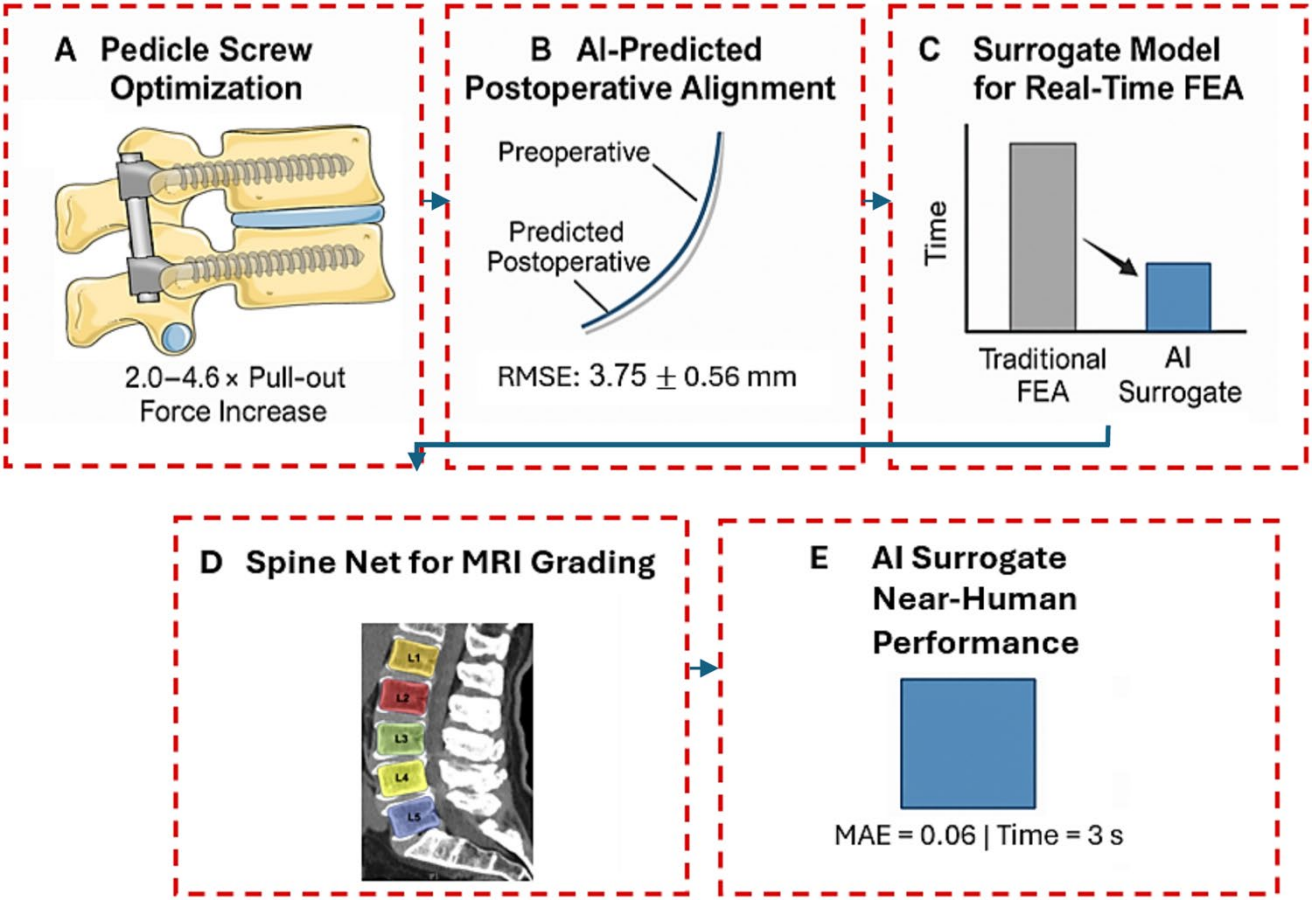
Innovative applications of artificial intelligence (AI) in enhancing spinal surgery outcomes

Figure 4 elaborates on the innovative applications of artificial intelligence in improving spinal surgery outcomes. In Section A (Pedicle Screw Optimization), it is demonstrated how AI can optimize the placement of pedicle screws, leading to a significant 2.0–4.6-fold increase in their pull-out force, which greatly enhances implant stability and longevity. Section B (AI-Predicted Postoperative Alignment) addresses AI’s capability in accurately predicting postoperative spinal alignment with a Root-Mean-Square Error (RMSE) of only 3.75 ± 0.56 mm. This high level of accuracy allows surgeons to plan more precisely for achieving optimal anatomical results. In Section C (Surrogate Model for Real-Time FEA), the most prominent advancement is showcased, where an AI-powered surrogate model drastically reduces the time required for Finite Element Analysis (FEA), while maintaining a comparable Mean Absolute Error (MAE) of 0.06 to traditional methods. This ability to perform FEA in near real-time significantly accelerates the surgical planning process. In the bottom-left of the figure, Section D (SpineNet MRI Grading) illustrates the application of AI in analyzing and grading spinal MRI images, which aids in more accurate and comprehensive diagnosis of the patient’s condition. It is worth noting, however, that the presence of two “L3” labels in this section is likely a typographical error, and the lower vertebra should be labeled L5. Finally, Section E (MAE 0.06 = 3 s/AI Surrogate/Near-human Performance) summarizes the remarkable efficiency of this system, emphasizing that the AI surrogate model can perform complex calculations with high accuracy (MAE 0.06) in just 3 s, achieving near-human performance. Overall, this figure clearly demonstrates that AI can revolutionize spinal surgical planning through enhanced precision, speed, and personalization. AI also bolsters preoperative risk identification, as demonstrated by Burns et al. [8], who developed an automated system to detect vertebral compression fractures on CT images. Achieving 95.7% sensitivity and a false-positive rate of 0.29 per patient, this tool identifies at-risk patients, enabling surgeons to adjust plans such as implant selection or screw placement to minimize intraoperative complications and enhance outcomes. Jamaludin et al. [35] developed SpineNet, a CNN-based framework that automatically grades spinal lumbar MRIs and localizes pathologies such as disc degeneration and canal stenosis. Achieving near-human performance across multiple gradings, SpineNet enhances preoperative diagnostics by providing rapid, accurate assessments, potentially reducing delays in treatment planning. Suzuki et al. [39] employed CNNs to detect lumbar spinal canal stenosis from plain radiographs, achieving an AUC of 0.90 and 82% accuracy in external validation. This tool facilitates early diagnosis in facilities without an MRI, potentially reducing treatment delays.

Addressing the computational burden of FEA, Atad et al. [8] introduced a neural network surrogate combined with projected gradient descent to calibrate finite element models of an L4–L5 intervertebral disc. Their method achieved a mean absolute error of 0.06 on synthetic data and reduced calibration time to under 3 s compared to days for traditional approaches. This rapid, accurate calibration could enable real-time biomechanical simulations, enhancing the feasibility of patient-specific surgical planning. In adolescent idiopathic scoliosis (AIS), AI is enhancing preoperative planning. Goldman et al. [12] reviewed its use in automatic measurement of Cobb angles and axial vertebral rotation, as well as curve classification, offering accurate tools to improve surgical preparation and decision-making for complex deformities. Barreto et al. [8] developed a 3D U-Net model for automated segmentation of lumbar vertebrae from MRI scans, achieving a Dice score of 0.84, followed by FEA to assess bone strength for personalized risk assessment. Sollmann et al. [89] review advanced imaging techniques for osteoporosis, emphasizing CT and MRI-based methods for bone quality assessment. These approaches, when integrated with AI, could enhance preoperative evaluations of vertebral strength, informing surgical strategies to mitigate fracture risks.

**Fig. 5:**
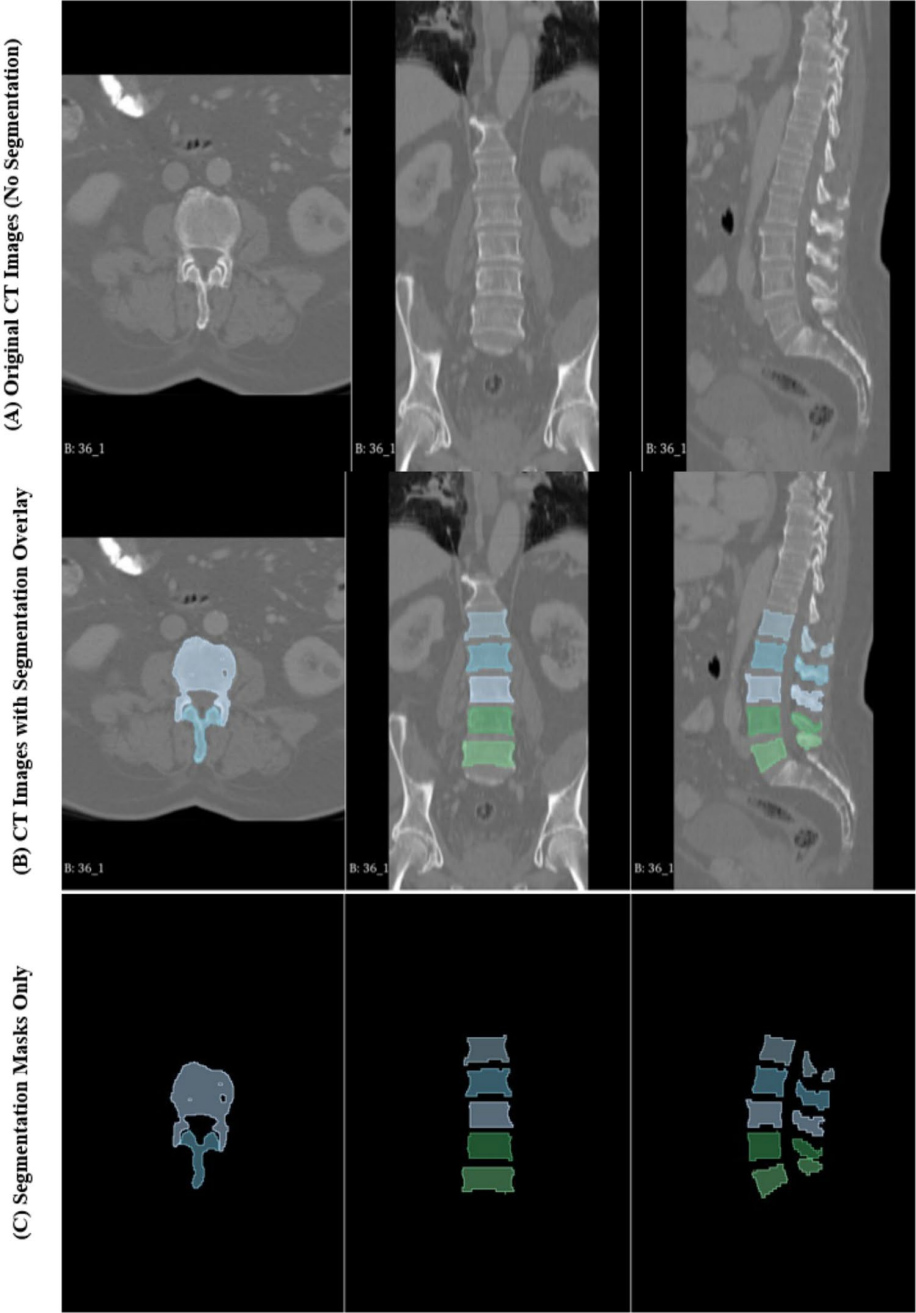
AI-assisted vertebral segmentation from CT imaging

Figure 5 demonstrates the role of artificial intelligence in segmenting spinal vertebrae for enhanced surgical planning. Row (A) displays the original CT scans in axial, coronal, and sagittal views without any segmentation. In Row (B), the segmented vertebrae are overlaid onto the original CT images, clearly identifying the anatomical structures and providing visual differentiation between vertebral levels. Row (C) presents only the segmentation masks, isolated from the background, offering a clearer view of the detected vertebrae. The color-coded masks enable accurate vertebral identification and spatial understanding of each segment, which is essential for surgical navigation, implant planning, and biomechanical analysis. This automated segmentation approach significantly reduces manual labor and enhances precision in preoperative modeling and simulation.

New studies complement these findings. Pedersen et al. [16] introduced PROPOSE, a machine learning tool predicting outcomes like walking distance improvement (AUC 0.88) and pain relief for lumbar spinal stenosis surgery, aiding shared decision-making. Schonfeld et al. [59] employed convolutional neural networks (CNNs) and large language models (LLMs) to predict postoperative complications in adult spinal deformity surgery, achieving an F1 score of 0.545 for pulmonary complications. Pellise et al. [89] leveraged radiomics and machine learning to predict mechanical complications, outperforming traditional radiographic measurements with an AUC − ROC of 0.75 compared to 0.71. Additionally, Karhade et al. [39] and Alassaf et al. [8] address implant failure risks, while Varghese et al. [71] enhance time-dependent modeling, all supporting the advancements in AI–FEA for surgical planning. When compared to traditional manual planning, which relies on surgeon experience and often achieves lower precision, AI–FEA tools offer a significant leap forward. For instance, Liang et al. [78] reported a 97.8% Dice similarity coefficient in their AI-driven segmentation models, far surpassing the accuracy of conventional methods, which often hover around 80–85%. Despite these advancements, limitations remain. Many of these models were validated on relatively small or homogeneous datasets, raising questions about their generalizability across diverse patient populations. Future research could focus on expanding these datasets and integrating additional variables, such as genetic or biomarker data, to further personalize surgical plans.

Table 3 summarizes pivotal studies combining AI–FEA across preoperative, intraoperative, and postoperative stages of spine surgery. It highlights diverse AI methods—like U-Net, PINNs, and AR—applied to tasks such as screw placement, spinal modeling, and risk prediction. Reported outcomes include high accuracy, reduced processing time, and improved surgical precision. These advancements demonstrate how AI–FEA integration is reshaping surgical planning with enhanced accuracy and efficiency.

**Table 3:** Summary of key studies on AI–FEA in spine surgery

Study	Year	Surgical phase	AI method	FEA application	Key outcome
Ao et al. [12]	2025	Intraoperative	Deep Reinforcement Learning	Screw placement planning	> 5% higher safety rates
Liang et al. [78]	2025	Preoperative	U-Net	Lumbar spine modeling	97.8% DSC, ~ 10 min generation
Zhang et al. [99]	2025	Postoperative	BPNN + XGBoost	Pressure ulcer risk	High performance, 1440 conditions, (*R*^2^) of 0.977
Atad et al. [8]	2025	Preoperative	NN surrogate + PGD	FE model calibration	MAE of 0.06 on synthetic data
Ahmadi et al. [4]	2025	Preoperative	PINNs	Material property prediction	94.30% accuracy
Luan and Morgan [34]	2025	Preoperative	Data-driven framework	Vertebral density-modulus	Unified density-modulus relationship
Berg et al. [12]	2024	Postoperative	Machine Learning	Outcome prediction	C statistic 0.82 for ODI
Soltani et al. [78]	2024	Postoperative	Predictive modeling	Fracture risk prediction	Accurate strength prediction, (*R*^2^) of 0.99
Zhang et al. [39]	2024	Intraoperative	AR	Spatial registration	Surface error 0.501 mm
Youssef et al. [92]	2024	Intraoperative	AR	Screw placement accuracy	93.1% clinical accuracy
Lin and Zhang [12]	2024	Intraoperative	PINNs	Spine deformation simulation	< 30 s per case, matches FEM
Zheng et al. [136]	2024	Preoperative	ANN + Probabilistic	Nutrient level analysis	Accurate IVD nutrient prediction
Singh et al. [16]	2024	Preoperative	Subject-specific	IVD degeneration modeling	Validated obesity-induced degeneration
Barreto et al. [8]	2024	Preoperative	3D U-Net	Bone strength assessment	Dice score 0.84, personalized risk assessment
Alassaf et al. [8]	2024	Preoperative	Machine Learning	Scoliosis correction planning	Time-dependent biomechanical evaluation
Pellise et al. [89]	2024	Preoperative	Radiomics + ML	Mechanical complication prediction	AUC − ROC 0.75
Schonfeld et al. [59]	2024	Preoperative	CNN/LLM	Complication prediction	*F*_1_: 0.545 for pulmonary complications
Pedersen et al. [16]	2024	Preoperative	Machine Learning	Outcome prediction	AUC 0.88 for walking distance
Muñoz-Moya et al. [40]	2024	Preoperative	PCA + Regression	IVD modeling	> 92% shape similarity
Phellan Aro (2024)	2024	Preoperative	Random Forests	Postoperative alignment	RMSE_pos 3.75 ± 0.56 mm
Grob et al. [39]	2024	Postoperative	Prediction models	Outcome prediction	AUC 0.70–0.72 for outcome prediction
Burstrom et al. [16]	2023	Intraoperative	Machine Learning	Screw placement assessment	High accuracy in placement evaluation
Nikkhoo et al. [59]	2023	Postoperative	AI–FEA	Adjacent segment biomechanics	Accurate stress prediction
Kok et al. [34]	2023	Intraoperative	Landmark-based	Load-bearing simulation	0.96 Dice, 10-min runtime
Jimenez et al. [16]	2023	Preoperative	Subject-specific	Cord decompression simulation	AUC 0.79–0.82
Fleps and Morgan [40]	2022	Postoperative	SVMs	Fracture risk prediction	< 8% MAE in vertebral strength
von Atzigen et al. [148]	2022	Intraoperative	Stereo Neural Network	Rod bending navigation	Marker-free AR navigation
Ma et al. [35]	2022	Preoperative	Machine Learning	Screw trajectory optimization	2.0–4.6× pull-out force increase
Zhang et al. [95]	2022	Intraoperative	CNN (KiUNet)	Vertebral stress analysis	92.8% 3D Dice index
Ahmadian et al. [5]	2022	Preoperative	ReconGAN (DCGAN)	Vertebral fracture risk	< 2% error in trabecular synthesis
Shruthi et al. [35]	2021	Intraoperative	Computational	Cervical disc kinematics	Bryan disc ROM slight increase
Caprara et al. [21]	2021	Postoperative	Automated workflow	FSU biomechanics	ROM matches literature, 2 h
Tajdari et al. [136, 137]	2021	Preoperative	Semi-automated	Spine curvature prediction	0.60–10.43% curvature error
Siemionow et al. [63]	[71]	Intraoperative	AR/AI	Surgical navigation	Feasible cadaveric navigation
Varghese et al. [71]	2018	Preoperative	Decision Tree	Pedicle screw pull-out strength	Improved instrumentation evaluation
Burns et al. [8]	2016	Preoperative	Automated system	Fracture detection	95.7% sensitivity

## Integration with smart wearables and IoT in spine recovery

The integration of smart wearables and Internet of Things (IoT) technologies has significantly reshaped the landscape of spine recovery and rehabilitation. Spine surgeons traditionally rely on validated instruments such as patient-reported pain levels, functional disability scores, radiographic findings, depression indices, and medication usage to assess treatment outcomes. However, the inclusion of Disability and Functional Outcome Measurements (DFOMs) objective metrics that assess physical function has added a new layer of clinical insight, increasingly becoming part of routine care. With the rise of wearable devices, particularly following the COVID-19 pandemic, there is growing demand for user-friendly and accessible technologies that provide real-time, quantifiable data on a patient’s spine health. These wearable tools—ranging from smartwatches to motion sensors and phone-based applications—offer clinicians the ability to track physical function, such as trunk movement, walking speed, and posture in real-world environments, outside the confines of a clinical setting. By integrating wearables with telehealth systems, healthcare providers can deliver more personalized and continuous care, especially during postoperative recovery.

Postoperative care is as critical as the surgical procedure itself, and recent advances in wearable technologies and Internet of Things (IoT) devices have transformed the monitoring and rehabilitation phases. Wearable sensors can continuously monitor spinal posture, detect asymmetries, and track progress during recovery [156]. These devices generate real-time biomechanical and physiological data that can be fed into AI algorithms to adjust physical therapy plans dynamically. Combined with AI, wearable devices can also predict complications such as infections, thrombosis, or poor implant integration by monitoring signs like local temperature, swelling, or movement patterns. Integration with smartphone-based dashboards allows patients and clinicians to maintain constant communication and feedback loops, improving adherence and satisfaction [63].

**Fig. 6:**
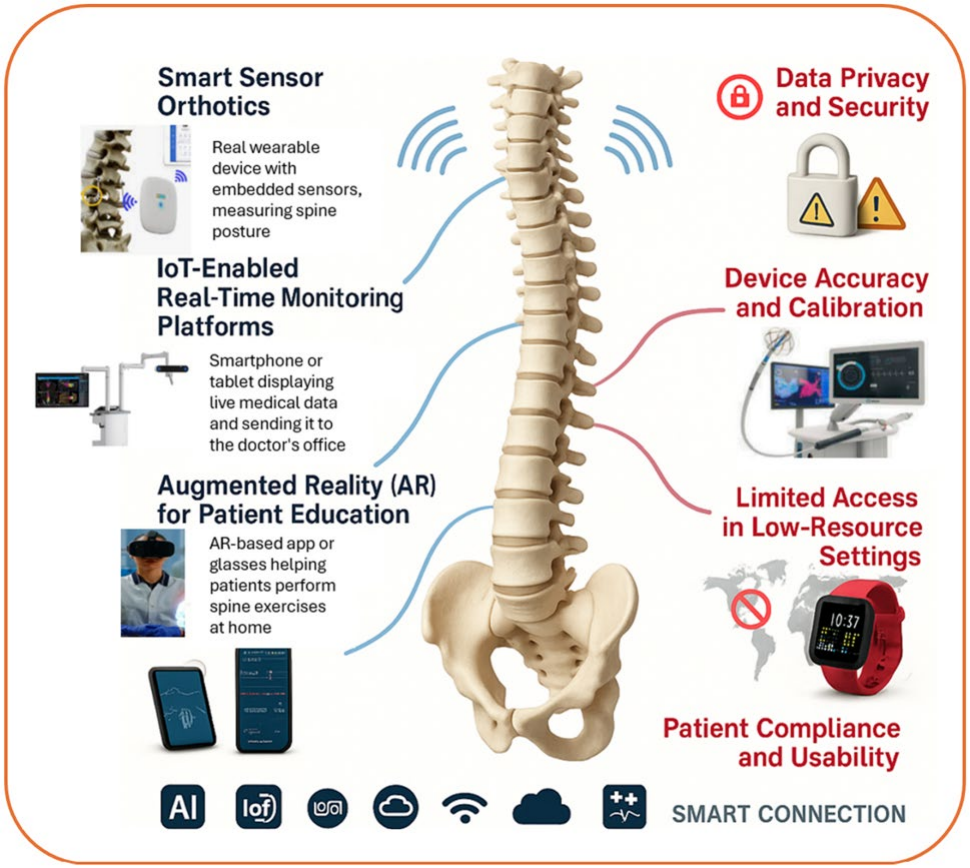
Integration of AI, IoT, and AR technologies in spine recovery and rehabilitation

Smart orthoses, powered by embedded sensors and actuators, represent another innovation. These assistive devices not only support weakened structures postoperatively but also respond to dynamic changes in motion and load. AI can optimize their support mechanisms in real time, providing customized mechanical reinforcement during movement. This biofeedback-based approach helps reduce dependence on manual rehabilitation alone and accelerates functional recovery [12, 99]. As these technologies mature, the next frontier is connecting wearables to hospital databases and digital twin frameworks. This would allow for a seamless transition of real-world patient behavior into virtual biomechanical simulations, facilitating proactive adjustments to treatment plans. Figure 6 illustrates the integration of advanced technologies such as Artificial Intelligence (AI), the Internet of Things (IoT), and Augmented Reality (AR) in spine recovery and rehabilitation. Key innovations include smart sensor orthotics for posture monitoring, IoT-enabled real-time platforms for continuous patient tracking, and AR tools to enhance patient education and engagement [12, 147]. These technologies aim to improve outcomes and personalize care, but several challenges remain.

These include concerns over data privacy and security in IoT devices, the need for accurate calibration, limited accessibility in low-resource settings, and ensuring patient compliance and usability. At the center of this ecosystem is the human spine, symbolizing the core focus of these technological advancements. Table 4 provides a comprehensive overview of recent studies that demonstrate how AI-enhanced smart wearables and IoT technologies are transforming spine rehabilitation. From real-time posture tracking and sensor-enabled spinal implants to personalized therapy through robotics and MEMS-based platforms, these innovations offer improved diagnostic accuracy, patient monitoring, and adaptive care. The integration of AR, LLMs, and digital health ecosystems shows strong potential in delivering accessible, efficient, and intelligent spine recovery solutions.

**Table 4:** Summary of key studies integrating smart wearables, IoT, and AI in spine rehabilitation

Author	Year	Method	Aim	Result
Calabrò et al. [12]	2025	Healthcare IoT-enabled body area networks (HIoT-BAN)	Improve sports injury rehabilitation detection using IoT in digital healthcare	HIoT-BAN improved detection accuracy, healthcare delivery speed, and error rate reduction
Boltaboyeva et al. [12]	2025	AI, IoT, LLMs with wearable sensors	Review adaptive rehabilitation systems for personalized care	AI and LLMs enhance personalization and automation in rehabilitation
Viswanathan et al. [147]	2025	SMART implants and sensor-based technology	Improve orthopedic outcomes in joint replacement	Enhanced intraoperative implant positioning and long-term monitoring
Yang et al. [89]	2025	IoT with MEMS sensors and PSO-SVM	Monitor adolescent physical training and rehabilitation	95% motion pattern recognition accuracy and < 250 ms response time
Mehmood, et al. [92]	2025	Wearables, AI, robotics, smart devices	Explore next-gen tech for patient care and rehabilitation	Automated diagnostics and virtual rehab systems improve accessibility
Aziz et al. [12]	2024	Review and conceptual framework using IoT devices in rehabilitation	To explore how wearable IoT devices enhance personalized rehabilitation by providing continuous monitoring and clinical support	IoT wearables improve patient monitoring, support clinical decisions, and raise ethical considerations in modern rehabilitation
Moghbelan et al. [95]	2024	Implementation of IoT and humanoid robotics framework (I-TROPHYTS)	To design a smart rehabilitation system using wearables and humanoid robots to track and adapt motor routines in real-time	The proposed system successfully monitored physical activity and adapted therapy using machine learning and edge computing
Ramachandran [39]	2024	Conceptual development of a connected digital health ecosystem	To establish a digital spine to enhance healthcare system efficiency, access, and decision-making through digital infrastructure	Digital ecosystems with AI and IoT can bridge healthcare access gaps and improve service delivery through real-time data flow
Lingampally et al. [40]	2024	Review of wearable robotic assistive devices with AI and ML integration	To evaluate wearable robotic technologies aiding musculoskeletal rehabilitation and assess their integration with AI for personalized therapy	AI-enhanced wearables offer affordable and accurate rehabilitation support, addressing fatigue and improving treatment consistency
Haddas et al. [21]	2023	Systematic review of wearable devices assessing DFOMs in spine care	To identify wearable devices used in spine-related DFOMs, analyze clinical studies, and explore integration into standard care	Wearable devices, primarily accelerometers, provide valuable real-time health data and support DFOM-based patient-specific decision-making in spine care
Szabo et al. [99]	2023	Literature review on sensor-based technologies in rehabilitation	To evaluate therapeutic advancements using sensor-based devices in medical rehabilitation	Sensor-based devices support monitoring and personalized therapy; their widespread use enhances rehabilitation effectiveness through adaptive technologies
Shah and Khang [34]	2023	Conceptual chapter on IoMT and healthcare transformation	To explore how IoMT and AI can enhance medical connectivity, automation, and reduce human dependency in healthcare	IoMT enables secure data exchange, boosts diagnostic accuracy, reduces costs, and accelerates digital transformation in healthcare services
Hodges and van den Hoorn [21]	2022	Narrative review and literature synthesis	To outline the current use of wearable sensors in low back pain (LBP) care and envision a future system integrated with AI and personalized monitoring	Wearable sensors show promise for personalized LBP treatment, but further development and integration are needed to realize full clinical utility
Kim et al. [71]	2022	Systematic review of SMART spinal implants	To evaluate available studies on SMART spinal implants and analyze their designs, applications, and clinical trends	SMART implants with sensors show potential for real-time load monitoring and fusion assessment, but clinical adoption is still limited

The ongoing advancement of wearable technology and artificial intelligence is opening new possibilities for predictive analytics in spine care. These tools can help develop individualized treatment plans by capturing longitudinal biomechanical data during daily activities—data that have traditionally been difficult to gather [21, 99]. Despite some limitations, such as the current dependence on laboratory settings and calibration challenges, wearable sensors are steadily becoming a vital part of spine health monitoring. Future directions point toward a more comprehensive, AI-powered ecosystem that not only monitors patients remotely but also supports decision-making, adherence, and tailored rehabilitation protocols based on real-time functional feedback.

## Educational impact: AI-based surgical simulation

The learning curve for spinal surgery remains steep, particularly for complex procedures, such as deformity correction or multilevel fusions. Traditional training methods often rely on cadaver labs, supervised practice, and time-consuming observations. However, AI-powered simulators and virtual reality (VR)/augmented reality (AR) platforms are reshaping surgical education [7]. These platforms use real patient data to simulate various surgical scenarios. AI tracks the trainee’s performance in real time, providing feedback on force application, tool trajectory, anatomical orientation, and procedural steps. As AI models are exposed to more surgical cases, they become more accurate in emulating real-world outcomes and mistakes [16].

Moreover, VR/AR simulations allow for safe repetition of high-risk maneuvers. Residents and fellows can practice multiple variations of a procedure without risking patient safety. When paired with haptic feedback and image-guided tracking systems, these simulators offer a near-realistic training environment. Integrating AI simulation into standardized residency curricula can also help equalize training quality across institutions. In developing regions, portable and low-cost VR-based trainers can supplement the lack of access to real surgeries or high-end equipment, supporting a more equitable healthcare workforce.

## Spine research acceleration through AI literature mining

The integration of artificial intelligence (AI), particularly natural language processing (NLP) and large language models (LLMs) has significantly accelerated the pace of spine research by enabling automated parsing of unstructured clinical data. Traditionally, radiological reports, operative notes, and consent forms in spine surgery were manually reviewed, making data extraction labor-intensive and prone to inconsistency [21, 63]. However, the emergence of NLP tools trained on vast corpora of clinical text, such as BERT, LLaMA-7B, and fine-tuned GPT models—has revolutionized this process. These tools can accurately identify key surgical events (e.g., incidental durotomy, wound drain usage, closure type), classify musculoskeletal pain characteristics (location, acuity), and categorize spine procedures based on informed consent notes with performance metrics often exceeding 90% accuracy, sensitivity, and predictive value [59].

**Fig. 7:**
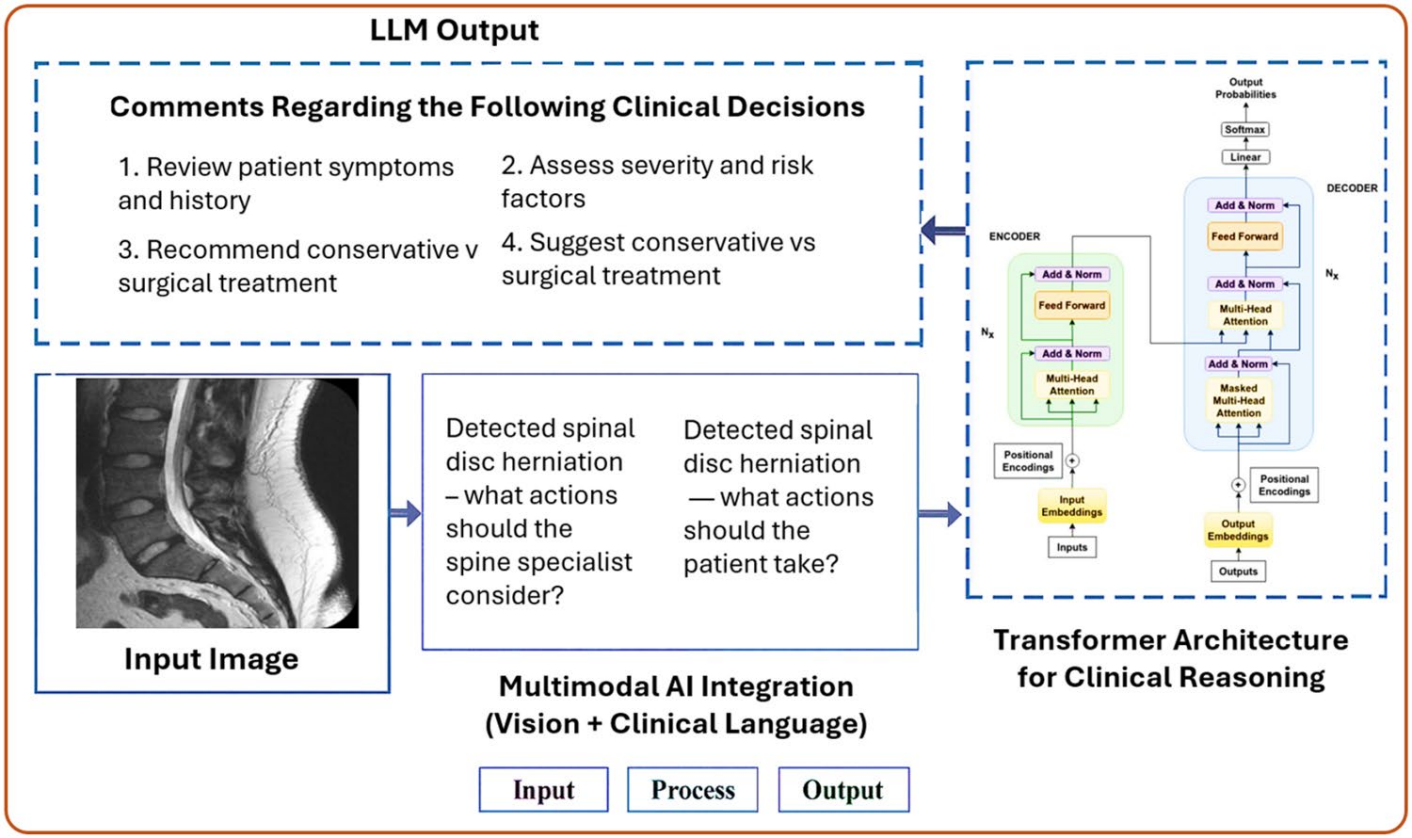
Multimodal AI system for spinal disc herniation diagnosis and decision-making. The system integrates medical imaging with clinical language using transformer-based architecture to generate targeted suggestions for both healthcare providers and patients

The surge in spine research has created a vast, fragmented body of knowledge. Manually reviewing hundreds of papers on spinal implants, surgical techniques, or biomechanics can be overwhelming. AI tools now facilitate scientific discovery by automating literature mining, trend analysis, and even hypothesis generation. Natural language processing (NLP) algorithms are used to extract information from thousands of research articles, identifying connections between techniques, outcomes, and patient groups. For example, AI can rapidly detect that patients over 65 with osteoporosis undergoing posterior lumbar fusion are more prone to screw loosening, based on cross-sectional analysis of literature and patient data [16]. Figure 7 demonstrates the role of multimodal artificial intelligence in spine research, particularly in supporting diagnostic reasoning and personalized clinical decision-making. The workflow starts with an input spinal MRI image, followed by natural language prompts regarding detected disc herniation. The questions—targeted toward spine specialists and patients—are processed using a transformer architecture that integrates vision and clinical language. The output includes AI-generated suggestions on clinical actions, such as evaluating patient symptoms, assessing severity, and determining conservative or surgical treatment options. This pipeline highlights the potential of AI to mine and synthesizes knowledge from imaging and text to accelerate spine research and improve outcomes.

Generative models can also summarize entire research domains, produce visual maps of scientific collaboration networks, and recommend articles based on relevance and novelty. This accelerates interdisciplinary research, enabling faster innovation across AI, FEA, materials science, and clinical disciplines. In research grant planning or systematic reviews, such AI tools reduce time and improve the objectivity of inclusion/exclusion criteria by classifying papers based on technical content and statistical strength [8]. These tools can also detect publication bias or redundant trials, guiding researchers to high-impact topics with unmet clinical needs [3]. Table 5 summarizes methods, aims, and outcomes across various applications, including diagnosis, treatment planning, risk assessment, and guideline interpretation.

**Table 5:** Overview of recent studies leveraging large language models (LLMs), multimodal AI, and NLP for spine research, clinical decision-making, and patient education

Author	Year	Method	Aim	Result
Eng et al. [35]	2025	Ten common rotator cuff repair questions were input into ChatGPT-3.5. Two surgeons evaluated the responses using JAMA Benchmark, DISCERN, and Flesch-Kincaid scores	To assess the quality of ChatGPT-3.5 responses to common patient questions about rotator cuff surgery	ChatGPT-3.5 lacked citation support, used complex language, and was rated as having only fair quality
Wang et al. [63]	2025	Twenty-one clinical questions from guidelines were input into ChatGPT-4o and 4o mini. Surgeon ratings and image analysis from 53 patients assessed accuracy, reliability, and readability	To compare ChatGPT-4o and 4o mini in diagnosing and managing lumbar disc herniation	Both models performed well, but their responses were hard to read. ChatGPT-4o was more consistent and detailed
Zhou et al. [40]	2025	Analyzed readability and DISCERN quality scores of AI-generated patient materials using ChatGPT and DeepSeek models for three spine surgeries	To evaluate the readability and reliability of AI-generated educational content for spinal surgeries	DeepSeek-R1 had the best readability. All models showed only fair quality due to lack of citation and personalization
Dong et al. [21]	2024	CNN-extracted MRI features and textual data were processed by a BERT-based LLM, validated with over 28,000 patient records and 514 external cases	To improve spine disorder classification by integrating MRI data and language models	Model identified 61 spinal disorder types with high accuracy and strong generalizability across datasets
Almekkawi et al. [8]	2025	Five clinical spine cases were given to LLMs and spine surgeons for comparison. MRI data was used to evaluate decision-making and radiological assessments	To compare LLMs and spine surgeons in clinical decision-making and image interpretation for spine pathologies	LLMs struggled with patient-specific details and had lower decision accuracy than surgeons (20% vs. 100%)
Wang et al. [35]	2024	Used EHRs and machine learning models (LSTM, XGBoost) to classify L5 and S1 radiculopathy based on symptom text and medical history	To develop and test AI models for diagnosing lumbar disc herniation using EHRs	LSTM model based on symptom text showed best performance with high accuracy and precision for L5/S1 diagnosis
Schonfeld et al. [59]	2024	CNN, LLM, and GWAS used to assess risks in ASD surgery using radiographs, clinical notes, and genetics on 209 patients	To predict surgical risks and complications in ASD patients using AI and genetic models	LLM outperformed CNN in predicting complications. GWAS identified significant genetic risk markers
Zaidat et al. [131]	2024	ChatGPT-3.5 and 4.0 answered 16 questions from NASS antibiotic guidelines; responses assessed for accuracy and confidence	To assess ChatGPT models â€™ ability to generate accurate antibiotic prophylaxis guidelines for spine surgery	GPT-4.0 was more accurate (81%) than GPT-3.5 (62.5%) and cited guidelines more frequently
Krebs et al. [35]	2023	Used NLP on MRI reports and logistic regression on clinical data to predict spine surgery need in referred patients	To develop a triage model for surgical referral decisions using NLP and clinical features	Clinical variables were stronger predictors than NLP; further validation needed
Subramanian et al. [95]	2023	ChatGPT was prompted with FAQs about minimally invasive spine surgery and its responses were evaluated	To explore the use of ChatGPT in answering patient questions in minimally invasive spine surgery	ChatGPT provided useful but general answers; further refinement is needed for patient-specific care
Vaid et al. [143]	2023	Fine-tuned LLaMA-7B on manually labeled clinical notes about musculoskeletal pain and tested model parsing accuracy	To assess large language models in extracting structured info from musculoskeletal pain notes	Model achieved high accuracy in location and acuity prediction; showed clinical potential
Shost et al. [124]	2023	Trained NLP classifier on consent forms labeled by surgery type using CPT codes; tested classification accuracy	To classify spine surgeries based on NLP analysis of informed consent documents	Model classified surgeries with 91% accuracy and high predictive values across procedures
Biswas et al. [16]	2023	Used XGBoost NLP models on operative notes to identify intraoperative elements like drains and sutures	To develop NLP models for detecting intraoperative events in lumbar spine surgery notes	Achieved > 91% accuracy, > 84% PPV, and high AUC; successfully identified intraoperative elements

AI-driven literature mining also offers a scalable and privacy-preserving approach to leverage historical data that was previously underutilized due to its unstructured nature. Studies have shown that NLP algorithms can extract detailed radiological findings from spine imaging reports, enabling weakly supervised learning of deep neural networks without relying on costly manual annotations [35, 95]. This approach enhances training efficiency and supports the development of predictive models for surgical outcomes, complication risks, and triage needs. Moreover, AI-generated structured annotations from existing clinical documents facilitate cohort identification for research, inform quality assessments in radiology, and support epidemiological studies of which contribute to more comprehensive and evidence-driven spine care [16].

Despite the transformative potential of AI in spine literature mining, certain limitations remain. General-purpose LLMs may hallucinate or provide overly generalized responses when not fine-tuned for medical domains, raising concerns about clinical reliability. Additionally, disparities in documentation styles and the lack of standardization in operative notes pose challenges for model generalization. Nonetheless, ongoing advancements in instruction-tuned models, the use of locally hosted AI systems for patient privacy, and adherence to machine learning reporting guidelines (e.g., TRIPOD, JMIR) are actively addressing these gaps. As a result, AI-enhanced literature mining is not only accelerating retrospective spine research but also laying the groundwork for predictive analytics and decision support systems in future clinical applications.

## Integration of AI-driven spine modeling for surgical planning and device optimization

**Fig. 8:**
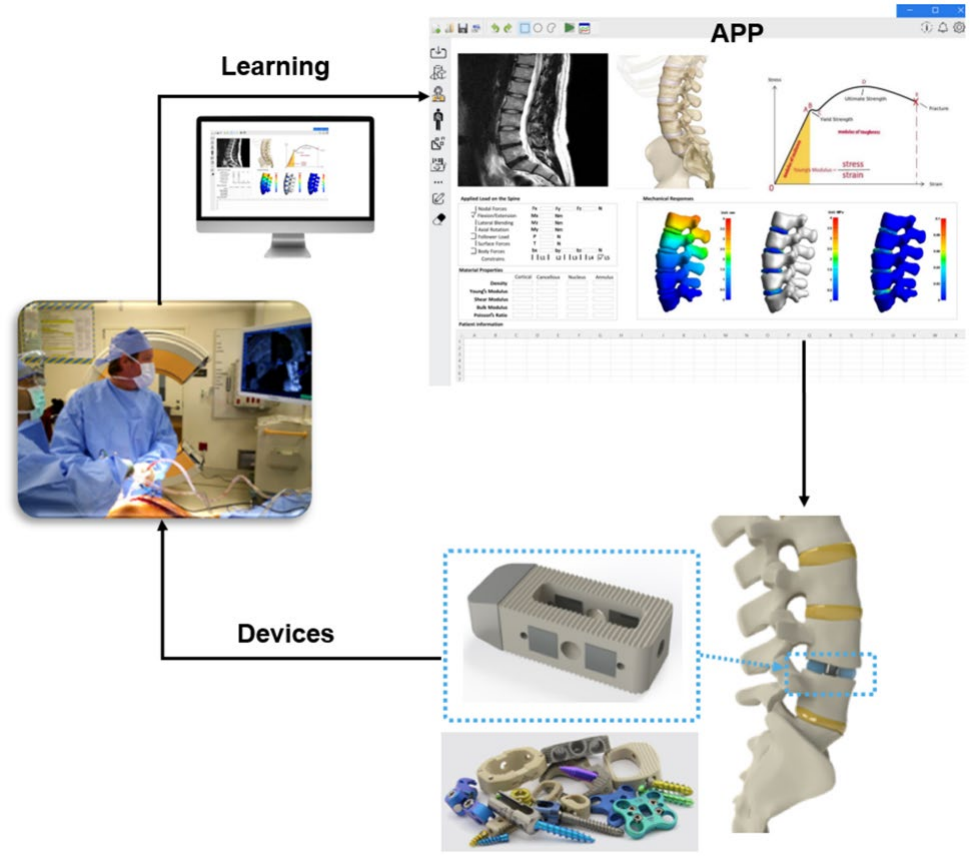
AI-driven spine care workflow integrating imaging-based simulations, personalized device design, and surgical application

The integration of artificial intelligence (AI) into spine surgery is redefining how clinicians approach surgical planning and implant development. Traditionally, implant design relied on static, generalized models that could not fully simulate the complex biomechanical behaviors of the spine under dynamic physiological conditions. With the adoption of AI-driven simulations, it is now possible to develop adaptable and patient-specific spine models that incorporate anatomical variations, loading conditions, and material properties. These models are not only more accurate but can also anticipate potential failure points and stress concentrations, ultimately enabling safer and more effective surgical interventions. Machine learning algorithms, particularly those trained on large datasets including medical imaging, patient demographics, and postoperative outcomes, have enabled predictive modeling that supports real-time decision-making in the operating room. AI enhances the personalization of care by adjusting surgical approaches and device configurations to suit each patient’s unique profile. Intraoperative data, including real-time imaging and monitoring, can be integrated into AI models to offer dynamic guidance to surgeons. This personalized and adaptive support enhances the precision of surgical maneuvers and improves patient outcomes, particularly in complex procedures involving spinal deformities or degenerative conditions. Figure 8 shows an AI-driven spine care workflow combining imaging-based simulations, personalized implant design, and surgical applications. Patient-specific spinal models guide surgical planning (APP), inform device customization (Devices), and support real-time decision-making during surgery (Learning), creating a closed-loop system for optimized outcomes. Table 6 presents a broad overview of contemporary studies that explore the integration of AI-driven methods in spine modeling, diagnostics, and surgical optimization. Several reviews and original studies (e.g., [21, 63, 77] discuss the role of machine learning, imaging analytics, and genomics in transforming personalized spine care. Emerging frameworks such as algorithm-hardware-ethics [54] and federated learning for cancer diagnosis [21] offer new pathways for robust and secure AI deployment.

**Table 6:** Summary of recent literature on the integration of AI, machine learning (ML), digital twins, and computational modeling in spine surgery, diagnostics, and treatment optimization

Author	Year	Topic	Application	Result
Kumar et al. [63]	2025	AI and ML in Contemporary Spine Care	Imaging, surgical planning, genomics, risk prediction	Highlights high-performing AI tools and precision platforms; addresses limitations in generalizability and data fragmentation
Yoganandan et al. [78]	2025	Patient-Specific Cervical Spine Finite Element Modeling	Spine biomechanics, FE modeling, military/civilian use	Emphasizes the need for individualized models in spine surgery; potential not fully realized
Han et al. [35]	2025	AI in Orthopedic Surgery	Pre/intra/postoperative planning and monitoring	Proposes 'algorithm-hardware-ethics' framework and calls for systematic review of AI deployment in orthopedic practice
Tiwari et al. [21]	2025	AI in Cancer Diagnostics and Treatment	Imaging, genomics, treatment planning, monitoring	AI enables early detection and personalization in oncology; future lies in federated learning and quantum AI
Lee et al. [76]	2024	Review of clinical task-based AI applications	Assess how AI transforms spinal imaging and patient care	AI improves image quality, enables better diagnosis, assists surgical planning, and personalizes treatment
Menta et al. [94]	2024	Narrative review	Evaluate technologies enhancing cervical spine trauma management	Technologies improve diagnostics, surgical precision, and recovery
Kalanjiyam et al. [66]	2024	narrative review	Summarize AI and machine learning applications in spine surgery	AI aids data analysis, treatment recommendations, and surgical planning
Bhimreddy et al. [8]	2024	Overview chapter	Present computational modeling, AR, and AI applications in spine surgery	Technologies assist preoperative planning, predictions, and education
Farahani [38]	2024	Review	Explore imaging data applications in surgery	Imaging enhances precision, safety, and recovery
Javanmard [62]	2024	Review article	Discuss AI's impact on healthcare and medical practice	AI improves diagnosis, treatment planning, and data analysis
Molina and Di Ieva [96]	2024	Chapter review	Explore AI, radiomics, and modeling in skull-base surgery	AI supports diagnosis, surgical planning, and procedural safety
Haleem et al. [34]	2023	Digital Twin + AI + IoT	To explore the use of digital twin technology in healthcare for personalized treatment planning	Digital twins combined with AI help in selecting optimal treatments and anticipating health issues
Paudyal et al. [63]	2023	AI in CT/MRI	To review AI applications in CT and MR imaging for oncology	AI enhances clinical efficiency and accuracy, especially in imaging for oncology
Song et al. [92]	2023	AI-based Deep Learning on X-ray	To automate spine segmentation and spinopelvic evaluation using X-rays	High accuracy in landmark detection and improved efficiency in diagnostics
Zeineldin et al. [12]	2023	AI-based Neurosurgery System	To develop an AI-guided system for neurosurgery to enhance precision and minimize invasiveness	Achieved high Dice score (0.87) and improved surgical guidance with AI
Esfahani et al. [16]	2023	ML using CT	To detect and classify cervical spine fractures using a machine learning model	Achieved 100% precision, recall, and specificity on RSNA dataset
	2022	AI in spinal deformity surgery	Improve safety and planning in adult spinal deformity	Enhanced surgical classification and outcome prediction
Firouzi et al. [39]	2022	AI + IoT + Cloud/Fog computing	Evaluate edge-fog-cloud integration in IoT	Optimized data processing and resource utilization
Kim et al. [59]	2022	AI-driven activity monitoring	Support post-surgery home recovery	Improved recovery tracking and reduced healthcare cost

Furthermore, AI-powered digital twins are being developed to virtually replicate the patient’s spine, enabling preoperative testing of different surgical scenarios. These virtual simulations allow surgeons to evaluate implant performance and optimize device placement before entering the operating room. Advanced AI models also assist in postoperative monitoring by predicting complications, such as implant loosening or adjacent segment disease. The impact of AI is especially significant in the development of next-generation implants that dynamically adjust to a patient’s lifestyle and loading conditions. Materials such as magnesium-based alloys, combined with machine learning-driven optimization, allow researchers to simulate how implants behave under various activities like walking or bending. These insights guide material selection and structural design before fabrication, promoting more durable, biocompatible, and functionally adaptive implants. As AI continues to evolve, its integration into the full surgical pipeline—from diagnosis to implant design and intraoperative navigation—will play a vital role in revolutionizing spine care and achieving truly personalized, outcome-driven treatment pathways.

## Standardization and interoperability

**Fig. 9:**
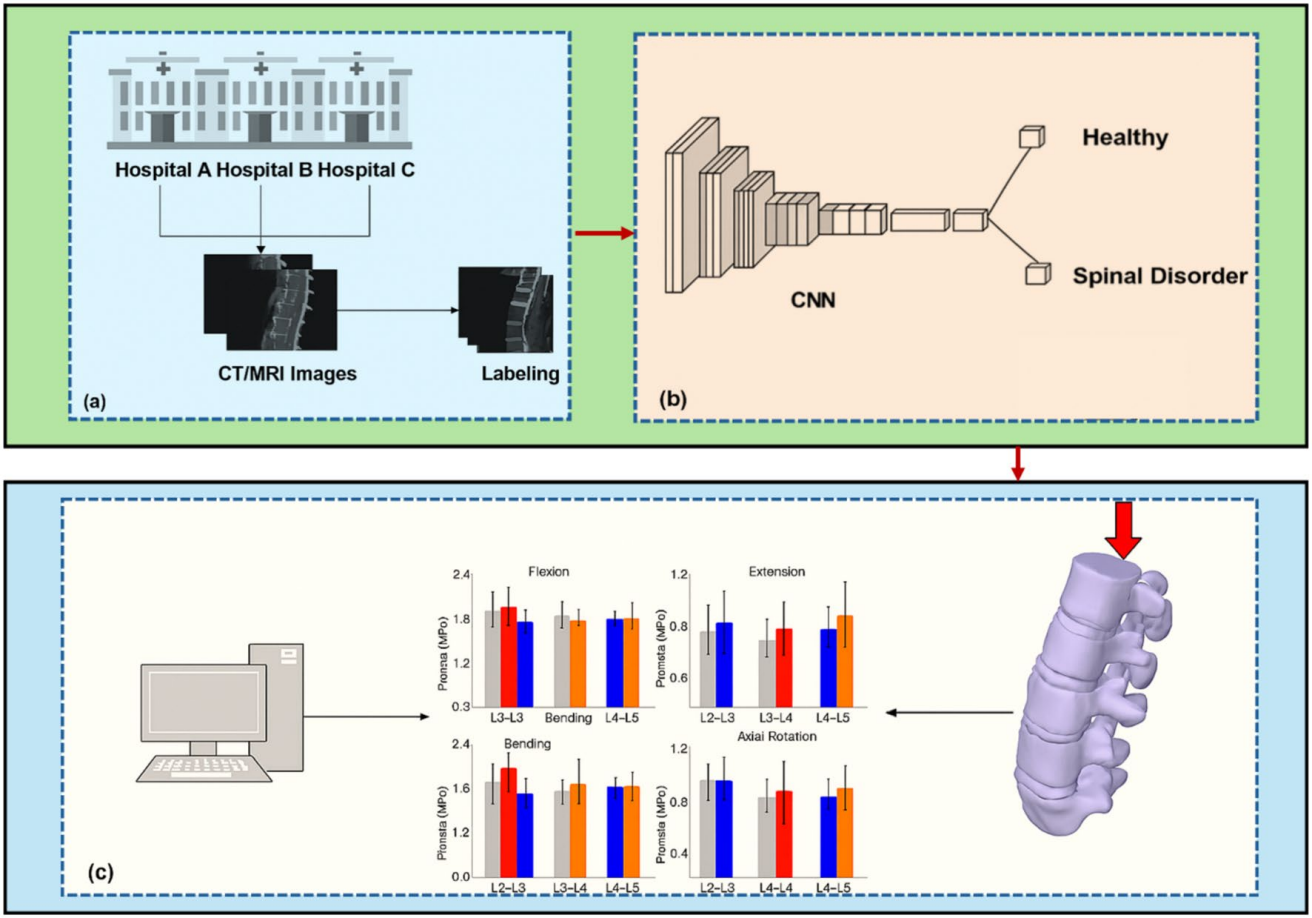
Standardized AI–FEA workflow for interoperable spine diagnostics. CT/MRI data from multiple hospitals are uniformly labeled and processed through convolutional neural networks (CNNs) to classify spinal health

For AI–FEA models to become clinically integrated, standardization is essential. Currently, most models are built on institution-specific data with proprietary software pipelines. This limits reproducibility and interoperability across hospitals and research centers. Efforts are underway to create universal protocols for spine segmentation, material mapping, and mesh generation. Open-source libraries and shared data repositories, such as the SpineWeb, TotalSegmentator, and MICCAI challenge datasets, are making standardized benchmarks more accessible [4]. These resources enable fair comparison of model performance and promote transparency. In addition, DICOM-compatible FEA pipelines and AI plugins for popular medical platforms (e.g., 3D Slicer, OsiriX) enable clinicians to use simulation results directly in treatment planning. Integrating AI–FEA tools with hospital information systems (HIS) and EHRs also enhances workflow efficiency and supports decision-making at the point of care. However, interoperability requires consensus on file formats, labeling conventions, and data pre-processing steps. Collaboration between regulatory agencies, software vendors, and medical societies is necessary to set standards that balance accuracy, computational demand, and usability (Fig. 9).

This multi-institutional pipeline emphasizes the critical need for harmonized imaging protocols and uniform labeling, ensuring that AI models perform consistently across diverse clinical settings [71, 119]. The AI-derived classifications are further enriched through biomechanical simulations using FEA under various loading conditions, such as flexion, extension, bending, and axial rotation. To maintain consistency, these simulations must apply standardized boundary conditions, material properties, and load parameters. The resulting quantitative outputs such as stress distribution across intervertebral segments offer valuable insights for clinical decision-making, but only when results are both interpretable and standardized [16, 95].

AI–FEA systems to become interoperable clinical tools, every component from data labeling and CNN training to FEA modeling and output interpretation must align with common frameworks, mesh standards, naming conventions, and anatomical landmarks. As these technologies evolve, building a shared ecosystem of tools, databases, and protocols will be essential for transforming spine diagnostics into a scalable, patient-specific solution [12].

## Toward autonomous spine surgery systems

As AI continues to evolve, the prospect of semi-autonomous or fully autonomous spine surgery becomes more realistic. Robotic platforms, such as the Mazor X and ROSA Spine, already provide AI-guided assistance in pedicle screw placement, with accuracy exceeding traditional methods. Future systems may combine real-time imaging, AI-driven anatomical recognition, and FEA-based force feedback prediction to allow robotic systems to perform tasks like decompression, discectomy, or fusion with minimal human intervention. Surgeons would shift from manual operators to supervisors, validating AI decisions and intervening only when unexpected conditions arise [12]. Autonomous systems could also enhance safety in remote or battlefield environments, where access to expert spine surgeons is limited. Coupled with 5G-enabled telesurgery platforms, AI-powered robotic systems could extend high-quality care across geographic boundaries. Nonetheless, substantial legal, ethical, and technological barriers remain. Ensuring real-time error detection, implementing safety overrides, and establishing liability frameworks are crucial before widespread adoption can occur.

**Fig. 10:**
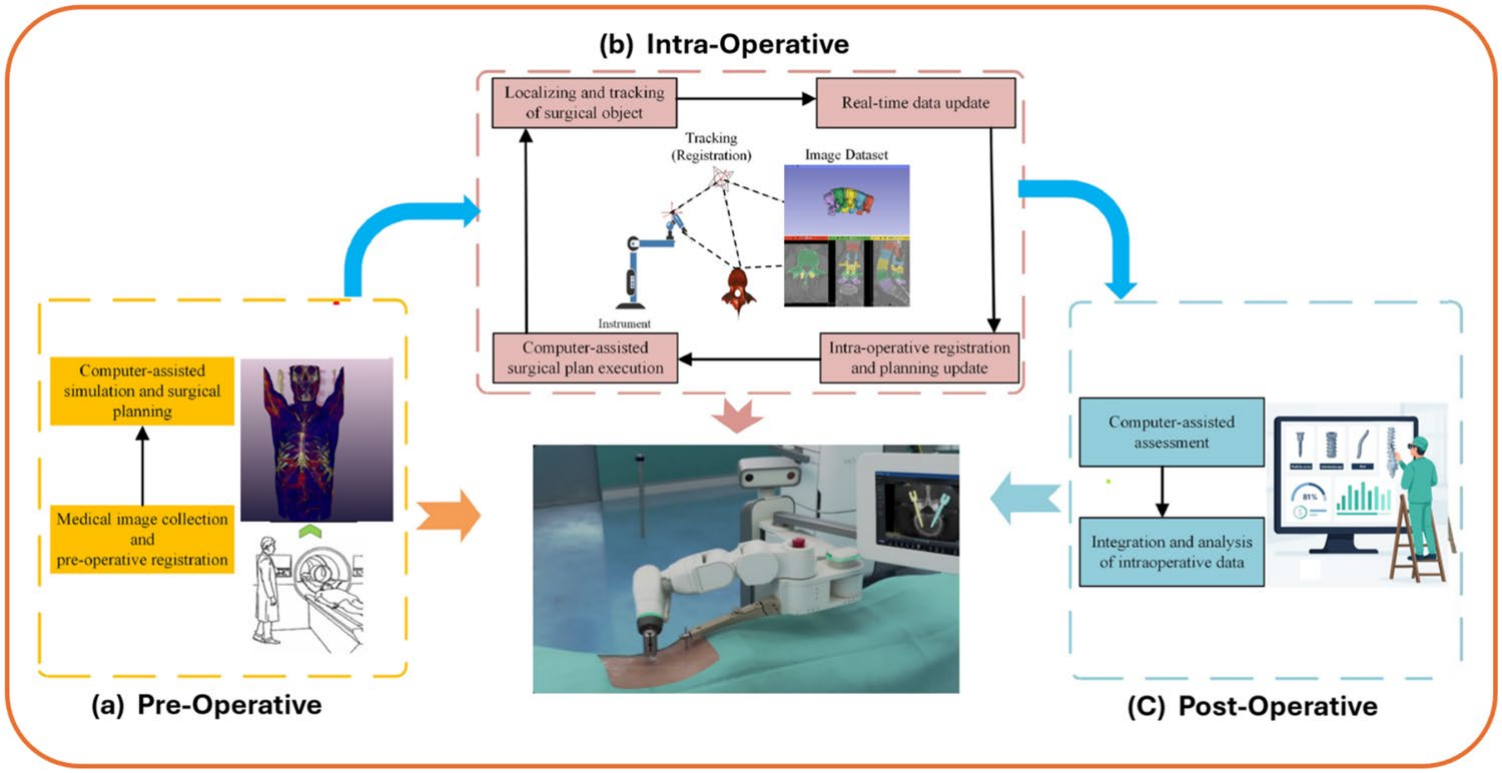
Overview of autonomous spine surgery workflow: **a** preoperative imaging and planning, **b** intraoperative navigation and robotic execution, and **c** postoperative assessment

Surgical planning and navigation are foundational elements of autonomous spine surgery systems. This stage initiates with the preoperative phase, where medical imaging technologies such as CT or MRI scans are utilized to construct patient-specific anatomical models. These models are then processed through computer-assisted simulation tools to develop precise surgical strategies tailored to the patient’s anatomy. This simulation not only aids in identifying the optimal trajectory and insertion points but also minimizes intraoperative risks. AI–FEA enhances precision in surgical execution. Ao et al. [12] developed SafeRPlan, a deep reinforcement learning tool for pedicle screw placement, achieving over 5% higher safety rates compared to conventional methods. This reduces screw misplacement risks, which Riewruja et al. [99] found occur in 5–10% of traditional cases, with their meta-analysis showing robot-assisted systems outperforming conventional, navigation, and augmented reality approaches in accuracy. Khalsa et al. [21] review the evolution of spinal robotics, noting current systems’ limitations to screw placement guidance but highlighting future potential through integration with advanced planning software and navigation, which could expand their utility in complex procedures. Wang et al. [124] reviewed robotic navigation in spine surgery, tracing its development since 2004 and evaluating current technologies. While primarily used for pedicle screw placement, they note rapid advancements that promise broader applications, enhancing surgical precision and efficiency. Tariciotti et al. [119] systematically reviewed AI applications in neurosurgical workflows, emphasizing intraoperative surgical assistance. They highlight how AI and ML, particularly neural networks and tree-based models, augment decision-making, reduce human errors, and enhance surgical precision across subspecialties, including spine surgery. Figure 10 illustrates the complete workflow of an autonomous spine surgery system across three main stages:

Furthermore, studies like Youssef et al. [92], Zhang et al. [39], Bui et al. [21], Siemionow et al. [63], Burstrom et al. [16], and Von Atzigen et al. [148] have advanced surgical planning and navigation through AR-assisted pedicle screw placement, spatial registration methods, AR/VR applications, and navigation improvements for rod bending. Youssef et al. [92] reported 93.1% clinical accuracy in AR-assisted screw placement, while Zhang et al. [39] achieved a surface registration error of just 0.501 mm for AR navigation. Bui et al. [21] highlighted AR/VR’s utility in surgical rehearsal and execution, and Siemionow et al. [63], Burstrom et al. [16], and Von Atzigen et al. [148] contributed to enhanced navigation for tasks like rod bending, preserving key metrics such as the 92.8% 3D Dice index from Zhang et al. [95].

These advancements collectively reduce intraoperative errors, streamline workflows, and enhance patient safety. Moreover, integrating AI–FEA with emerging technologies like robotic surgical systems and augmented reality (AR) holds promise for further precision and efficiency. Table 7 summarizes recent advancements in surgical planning, navigation, and assessment technologies across various spine and cranial procedures. For instance, combining AI–FEA with robotics could automate tasks like screw insertion, while AR overlays building on work like Youssef et al. [92] could provide real-time 3D visualizations of AI-generated models. However, challenges persist, including high computational demands and the need for broader validation, as noted in controlled studies like Zhang et al. [95]. Additionally, seamless integration into existing operating room systems requires addressing resource constraints and ensuring compatibility with current workflows. Table 3 outlines the synergistic outcomes of combining AI–FEA with AR and robotics across the surgical continuum, highlighting their contributions to preoperative, intraoperative, and postoperative phases. Overview of the synergistic contributions of AI–FEA and AR/robotics in spine surgery, detailing AI–FEA contributions, AR/robotics enhancements, and resulting outcomes across preoperative, intraoperative, and postoperative phases.

**Table 7:** Summary of key studies on AI-driven surgical planning, navigation systems, and outcome assessment in spine and related surgeries

Author	Year	Method	Aim	Result
Fleps and Morgan [40]	2025	Support Vector Machines (SVM)	Predict vertebral strength using FEA simulation	Achieved MAE < 8%; enables precise fracture risk assessment
Caprara et al. [26]	2022	Automated FEA Workflow	Create patient-specific FEA models of spinal units	Completed within 2 h; useful for fast evaluation of spinal stability
	2025	Multivariable Predictive Models	Predict patient-reported outcomes (PROs) after lumbar spine surgery	Key predictors: age, pathology, comorbidities, prior surgeries, hospital stay
Ghogawala et al. [46]	2025	Randomized-Controlled Trial	Compare ventral vs dorsal cervical surgery using PROs	Ventral had higher complication rate (48% vs 24%); similar PROs at 12 months
	2025	Step Count Analysis	Assess effect of early walking on pain and disability outcomes	≥ 3500 steps/day improved back/leg pain and disability outcomes
	2025	Registry Analysis (PROMIS data)	Study effect of complications on PROs in a large patient cohort	Complications linked to lower physical and mental health scores
Park et al. [107]	2025	Socioeconomic Data Analysis	Examine impact of socioeconomic factors on surgery satisfaction	Lower SES → reduced satisfaction and likelihood of reaching MCID
Mooney et al. [98]	2025	Comparative Outcome Study	Compare inpatient vs outpatient lumbar decompression surgeries	Outpatient surgery was non-inferior in terms of satisfaction and PROs
Isikay et al. [34]	2024	3D visualization and reality technologies	Enhance surgical training, planning, and navigation in skull-base neurosurgery	Improved surgical accuracy, planning, and education through patient-specific models
Schmidt et al. [21]	2024	Stereotactic navigation-guided endoscopic spine surgery	Describe principles and applications of navigation in full-endoscopic spine surgery	Navigation reduces guesswork and complexity, improves surgical outcomes
Najera et al. [21]	2024	Mixed Reality (MxR)	Evaluate the efficacy of MxR for AVM surgical planning and navigation	Facilitated arterial feeder identification; reduced surgical time and blood loss
Wilson et al. [16]	2024	Image-guided navigation	Review historical development and future directions of spine surgery navigation	Enhanced surgical accuracy and reduced revision surgeries
Sanchez-Sotelo et al. [40]	2024	Mixed-reality surgical navigation with cadaveric model	Validate MR accuracy in glenoid pin placement for shoulder arthroplasty	Mean deviation in placement was minimal, confirming high accuracy
Tzelnick et al. [59]	2023	Narrative Review	Outline current state and future of surgical navigation in skull-base surgery	Enhanced 3D anatomical orientation and intraoperative guidance
Aghaloo et al. [2]	2023	Systematic Review and Meta-analysis	Evaluate accuracy and survival of guided vs navigation implant surgery	Implant placement with both methods showed high survival and similar accuracy
Han et al. [16]	2023	Optimization & Simulation	Propose viewpoint optimization for optical tracking in orthopedic surgery	Maintained navigation consistency under spatial variations
Qin et al. [12]	2023	Dual-Robot Navigation System	Improve preoperative planning and intraoperative navigation with collaborative robots	Enhanced navigation precision and minimized surgical uncertainties
Park et al. [35]	2023	Prospective Study	Assess feasibility of patient-specific 3D navigation in gastric surgery	3D guidance was feasible with acceptable operative outcomes
Sozzi et al. [12]	2022	Brainlab Vector Vision 3.0 navigation	Evaluate accuracy of surgical navigation in mandibular reconstruction	Mean discrepancy from 0.66 mm to 1.46 mm; protocol considered accurate and versatile
Otomo et al. [34]	2022	CT-based navigation system	Review CT navigation in spine surgery	Effective for minimally invasive spine stabilization; enables precise patient positioning
Gubian et al. [40]	2022	CT navigation with 3D-trajectory planning	Evaluate screw placement accuracy	Screw placement acceptable, but deviations from plan observed
Tang et al. [40]	2022	Mixed Reality with surgical navigation	Evaluate feasibility and accuracy for maxillofacial tumor surgery	Mean deviation 1.68 ± 0.92 mm; accurate and feasible approach
Cheng et al. [21]	2022	Navigation-aided vs. conventional surgery	Evaluate reduction accuracy in ZMC fractures	No significant difference in translational or rotational errors
Chan et al. [16]	2022	Systematic review of VSP in maxillary reconstruction	Synthesize evidence on virtual surgical planning	High heterogeneity; lack of standardized outcomes; further research needed
de Geer et al. [12]	2022	Systematic review of registration methods	Review registration techniques in mandibular surgery	Four main methods identified; trade-off exists between accuracy and usability

Table 8 outlines the synergistic integration of Artificial Intelligence (AI)-driven Finite Element Analysis (FEA) with Augmented Reality (AR) and robotic technologies across the surgical workflow. In the preoperative phase, AI–FEA helps simulate patient-specific spinal mechanics, while AR and robotics enhance visualization and screw path planning. During the intraoperative phase, real-time data refine FEA predictions, while robotic systems ensure precise instrument control and AR assists with surgical navigation. In the postoperative phase, FEA models and AI monitor implant integration and patient outcomes, with robotic tools supporting rehabilitation. This synergy enables personalized, data-driven, and error-reduced spine surgery. In the intraoperative phase, surgical navigation systems take center stage by integrating real-time tracking of instruments and anatomy with the pre-planned surgical strategy. Through continuous updates of the surgical field using intraoperative registration and data fusion surgeons are guided with high precision during the procedure. Robotic arms, equipped with sensors and guided by computer vision, execute these plans under human supervision or semi-autonomous control, significantly improving accuracy and repeatability. Robotic platforms are a cornerstone of autonomous systems, offering unparalleled precision for surgical tasks. These systems enhance intraoperative navigation and execution, minimizing human error and improving the accuracy of procedures like pedicle screw placement.

**Table 8:** Emerging technologies—AI–FEA synergies with AR/robotics

	AI–FEA contribution	AR/robotics contribution	Synergistic outcome
Preoperative	AI-driven FEA simulates patient-specific spinal anatomy and biomechanics Optimizing implant positioning and trajectory	AR enables 3D visualization of spinal anatomy with virtual planning overlays Robotics offer precise preoperative simulation of screw pathways	Personalized preoperative surgical plans for optimal accuracy
Intraoperative	Al models update FEA predictions based on intraoperative data Identifies risk zones for hardware failure	Robotic arms maintain precise surgical incisions AR offer visual guidance and accurate depth perception	Data informed surgical execution with reduced risk of error or misplacement
Postoperative	FEA models predict implant integration and stress distribution Al assesses long-term outcome risks	Robotics support assisted recovery therapy	Tailored rehabilitation and early complication prediction

## Postoperative assessments and predictive modeling

AI-driven finite element analysis (AI–FEA) is critical for enhancing postoperative care, offering personalized predictions of biomechanical risks, complications, and functional recovery. By integrating AI with FEA simulations and clinical data, these tools enable clinicians to create tailored rehabilitation strategies and proactively manage postoperative issues. A primary application is the prediction of specific biomechanical complications. For instance, models can accurately forecast fracture risk, which informs decisions on weight-bearing restrictions and activity modifications for vulnerable patients. AI–FEA is also used to predict stress in adjacent spinal segments after fusion and to assess vertebral strength in metastatic spines, thereby enhancing risk assessment for implant failure or secondary fractures. Furthermore, AI–FEA excels in modeling soft-tissue stress and strain, which allows for the precise risk classification of pressure ulcers in patients with spinal cord injuries. This predictive capability enables early interventions, such as using pressure-relieving mattresses or adjusting repositioning protocols, to mitigate risk.

Beyond biomechanical failures, AI–FEA models are increasingly used to predict functional recovery and patient-reported outcomes. Studies have successfully validated models that predict the Oswestry Disability Index (ODI) and pain scores following spinal fusion, helping to set realistic recovery expectations and personalize rehabilitation protocols. This approach provides personalized prognostic insights, enabling tailored recovery plans and improved patient counseling. Other models can identify high-risk patients for hospital readmission or forecast curve progression in conditions like adolescent idiopathic scoliosis (AIS), giving clinicians valuable data for long-term monitoring and intervention planning. The various data sources that inform these predictive models are summarized in Table 9. This highlights the multidisciplinary nature of modern spine care, which combines clinical data, imaging, and patient-reported outcomes to support personalized treatment and long-term management.

**Table 9:** Overview of imaging, computational, and patient-reported data modalities in spine research and clinical application

Modality	Main applications	Advantages	Limitations
EHR/registries	Clinical decision support, outcome prediction	Extensive real-world data, longitudinal tracking	Data heterogeneity, unstructured formats
Wearables/IoT	Monitoring motion/posture, remote rehabilitation	Real-time, continuous monitoring of health metrics	Device calibration needs, patient adherence
Omics	Genomic risk assessment, multiomics integration	Mechanistic insights, personalized treatment strategies	Complex analysis, ethical/privacy risks
Computed tomography (CT)	Detection of fractures and spinal pathologies	Rapid imaging with high sensitivity for fractures	Radiation exposure, limited soft-tissue detail
Multimodal AI	Integrated data analysis across clinical sources	Improved diagnosis, personalized interventions	Data fusion complexity, standardization issues
PROs (patient-reported outcomes)	Evaluating recovery, patient satisfaction, QoL	Patient-centric care, better quality assessment	Variation in reporting, data collection burden

Despite these advancements, limitations remain. Many current models rely on retrospective data from specific cohorts, which may introduce bias and reduce their accuracy across diverse patient populations. Future work must focus on validating these predictive tools in larger, prospective studies to ensure their reliability and clinical utility.

AI–FEA also excels in predicting specific postoperative complications. Zhang et al. [99] utilized a hybrid model combining backpropagation neural networks (BPNN)—a type of artificial neural network and Extreme Gradient Boosting (XGBoost), a decision tree-based ensemble algorithm, to predict soft-tissue stress and strain in spinal cord injury patients, achieving a coefficient of determination (*R*^2^) of 0.977 on test data. This high accuracy supports precise risk classification for pressure ulcers based on predicted strain levels, which could enable early interventions (e.g., pressure-relieving mattresses or repositioning protocols) to mitigate risk. However, the study focuses on predictive accuracy rather than direct clinical validation of ulcer reduction. Nikkhoo et al. [59] applied AI–FEA to predict biomechanical responses in adjacent segments post-fusion, achieving accurate stress predictions, while Soltani et al. [78] used CT-based FEA to predict both vertebral strength and stiffness in metastatic spines with (*R*^2^) of 0.99 on specimen specific calibration, enhancing fracture risk assessment. Similarly, Hasanpour et al. [39] applied machine learning to FEA data to predict adjacent vertebral fractures following vertebroplasty, emphasizing AI’s role in evaluating biomechanical risks post-surgery. Additionally, Muñoz-Moya et al. [40] used principal component analysis and regression to model intervertebral disc (IVD) mechanics, achieving over 92% shape similarity to ex vivo data. This accurate modeling aids in assessing disc behavior after surgery, supporting strategies to prevent disc-related complications like degeneration.

**Fig. 11:**
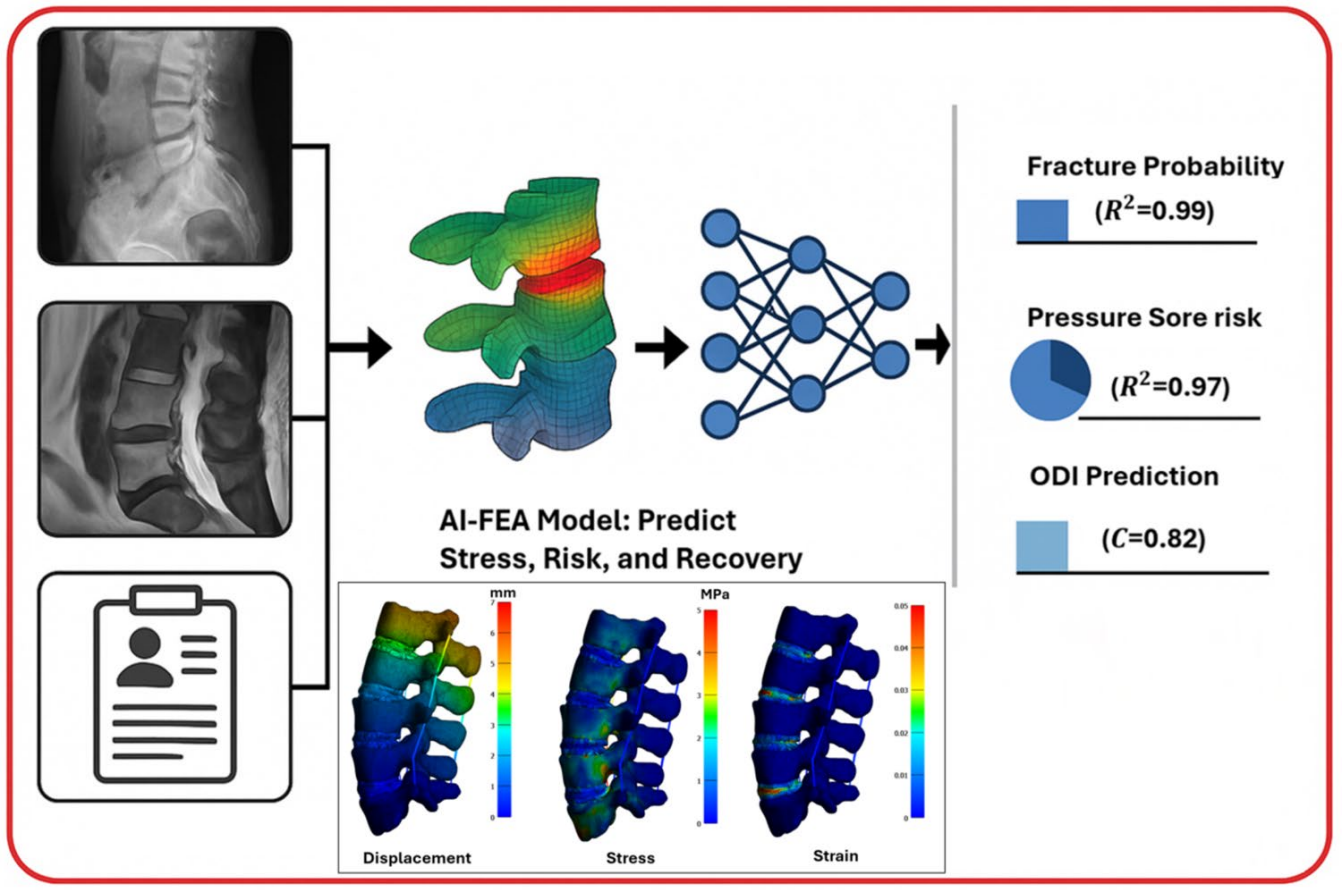
Workflow of AI–FEA-based postoperative risk prediction. Patient imaging and clinical data are used to simulate biomechanical stress and train predictive models, providing estimates for key outcomes, such as fracture risk, pressure sore likelihood, and functional recovery after spine surgery

Beyond complications, AI–FEA also predicts functional recovery. Grob et al. [39] validated prediction models for Oswestry Disability Index (ODI) and pain scores after spinal fusion, achieving AUCs of 0.70–0.72. These models assist in setting realistic recovery expectations and personalizing rehab protocols. Similarly, Berg et al. [12] applied machine learning to predict disability and pain for 12 months following lumbar disc herniation surgery. Their model achieved a *C* statistic of 0.82 for the Oswestry Disability Index, reflecting strong predictive accuracy. Validated across multiple regions, this approach provides personalized prognostic insights, enabling tailored rehabilitation plans and improved patient counseling post-surgery. Kalagara et al. [12] developed machine learning models to predict hospital readmissions following lumbar laminectomy, achieving over 79% accuracy using predischarge variables. This tool supports postoperative care by identifying high-risk patients, enabling targeted interventions to reduce readmission rates. AI also aids in predicting postoperative outcomes in AIS. Goldman et al. [12] highlighted models that forecast curve progression with an average accuracy of 85.4%, providing clinicians with valuable insights for monitoring and planning subsequent interventions. Despite these advancements, limitations remain. Figure 11 illustrates the role of AI–FEA models in predicting patient-specific postoperative risks following spine surgery. By integrating imaging data and clinical records, AI-enhanced finite element simulations estimate biomechanical parameters, such as stress, strain, and displacement. These biomechanical features are then fed into machine learning models to predict critical postoperative outcomes, including fracture probability, pressure sore risk, and functional recovery measured by the Oswestry Disability Index (ODI). This approach enables early identification of complications and supports personalized rehabilitation planning.

Additionally, the computational demands of real-time FEA simulations pose a barrier to widespread clinical use. Future research should focus on prospective validation across varied patient groups and explore integration with technologies like wearable sensors or AI-guided rehabilitation apps to enhance real-time monitoring and patient engagement. Figure presents an overview of an AI-integrated finite element analysis (AI–FEA) framework used for predicting postoperative risks and recovery outcomes in spine surgery. The process begins with patient-specific medical imaging and clinical records, which are fed into a biomechanical simulation model. The AI–FEA system analyzes spinal stress distributions and uses machine learning to assess potential postoperative complications, such as fracture risk, pressure sore development, and functional disability. These predictive insights enable clinicians to make data-driven decisions for personalized rehabilitation planning, enhancing both short- and long-term patient outcomes.

## Challenges and future directions

The adoption of AI–FEA in spine surgery faces significant implementation barriers, primarily related to computational and financial demands. The high computational complexity of these models requires substantial processing power, which may not be available in all clinical settings. This contributes directly to the financial burden of implementation, as the acquisition and maintenance of advanced intraoperative imaging, navigation systems, and specialized hardware are capital-intensive. While these technologies can lead to long-term savings by reducing revision surgeries, the significant upfront cost poses a major barrier for many institutions.

A second major hurdle involves model validation and clinical integration. Many current AI models are trained on limited or homogeneous datasets, raising concerns about their generalizability across diverse patient populations. This necessitates investment in large-scale, multi-institutional data collection and validation studies. Furthermore, there is a steep learning curve for surgeons and staff, with proficiency often requiring 20–30 cases. Integrating the complex outputs of AI–FEA into existing surgical workflows is challenging and can initially increase procedure times, highlighting the need for comprehensive and accessible training programs. Beyond logistical and financial hurdles, significant ethical and regulatory challenges remain. Ensuring patient data privacy is paramount, and decentralized methods like federated learning are being explored to train models without sharing sensitive patient data. Furthermore, establishing clear regulatory pathways and standardized protocols for the clinical approval of AI–FEA tools is essential for their safe and widespread adoption.

Future directions to overcome these barriers must focus on practical, multi-pronged strategies. This includes enhancing cost-effectiveness through innovative purchasing or leasing models, investing in comprehensive simulation-based training to reduce the learning curve, and promoting further research to strengthen the clinical and economic evidence for these technologies. Integrating AI–FEA with enabling platforms like robotics and augmented reality offers promising avenues for creating more powerful and intuitive systems. Ultimately, overcoming these multifaceted barriers will depend on collaborative efforts between clinicians, engineers, and policymakers to foster innovation while ensuring patient safety and equitable access to these transformative technologies.

## Conclusion

The integration of artificial intelligence (AI) and finite element analysis (FEA) represents a significant paradigm shift in spine surgery, enabling personalized, predictive, and data-driven interventions. AI-enhanced FEA allows for rapid, high-fidelity biomechanical simulations that support surgical planning, intraoperative navigation, and postoperative risk assessment. These models empower clinicians to tailor treatment strategies based on individual anatomy, physiological response, and predicted complications—ultimately improving outcomes and reducing procedural risk. From optimized pedicle screw placement to advanced intervertebral disc modeling, the clinical impact of AI–FEA is evident. Emerging techniques such as physics-informed neural networks for material prediction and rapid surrogate model calibration suggest a future where AI–FEA delivers real-time, patient-specific simulations. Many existing models rely on limited or homogeneous datasets, which may restrict their generalizability to diverse patient populations. Real-time simulations still demand high-performance computing resources not readily available in all clinical settings. Moreover, regulatory uncertainties, lack of standardized workflows, and ethical concerns surrounding data privacy and algorithmic transparency continue to pose barriers to widespread clinical adoption. To overcome these challenges, future efforts must focus on prospective clinical validation, broader multi-institutional data integration, and the development of explainable, transparent AI systems. Incorporating wearable sensors, smart implants, and electronic health record connectivity will further enhance real-time feedback and adaptive decision-making. Ultimately, realizing the full potential of AI–FEA in spine surgery will require close collaboration among clinicians, biomedical engineers, computer scientists, and policymakers. If successfully implemented, AI–FEA could become a foundational technology in next-generation, intelligent spine care systems—improving safety, efficiency, and patient outcomes at scale.

## Data Availability

No datasets were generated or analyzed during the current study.
